# Structural Speciation of Ti(IV)-(α-Hydroxycarboxylic Acid) Complexes in Metabolism-Related (Patho)Physiology—In Vitro Approaches to (Pre)Adipocyte Differentiation and Mineralization

**DOI:** 10.3390/ijms241411865

**Published:** 2023-07-24

**Authors:** Olga Tsave, Catherine Iordanidou, Antonios Hatzidimitriou, Maria P. Yavropoulou, Eva N. Kassi, Narjes Nasiri-Ansari, Catherine Gabriel, Athanasios Salifoglou

**Affiliations:** 1 Laboratory of Inorganic Chemistry and Advanced Materials, School of Chemical Engineering, Aristotle University of Thessaloniki, 54124 Thessaloniki, Greece; tsaveolga@auth.gr (O.T.); theskiordani@yahoo.com (C.I.); katerinagabriel@yahoo.gr (C.G.); 2 Laboratory of Inorganic Chemistry, School of Chemistry, Aristotle University of Thessaloniki, 54124 Thessaloniki, Greece; hatzidim@chem.auth.gr; 3 Endocrinology Unit, 1st Department of Propaedeutic and Internal Medicine, Medical School, National and Kapodistrian University of Athens, 11527 Athens, Greece; myavropoulou@med.uoa.gr (M.P.Y.); ekassi@med.uoa.gr (E.N.K.); 4 Department of Biological Chemistry, Medical School, National and Kapodistrian University of Athens, 11527 Athens, Greece; nnasiri@med.uoa.gr

**Keywords:** titanium, mineralization, bioavailable titanoforms, insulin mimesis, adipocyte differentiation, adipogenicity and osteogenicity, metallopharmaceuticals

## Abstract

The prospect of developing soluble and bioavailable Ti(IV) complex forms with physiological substrates, capable of influencing (patho)physiological aberrations, emerges as a challenge in the case of metabolism-related pathologies (e.g., diabetes mellitus 1 and 2). To that end, pH-specific synthetic efforts on binary Ti(IV)-(α-hydroxycarboxylic acid) systems, involving natural physiological chelator ligands (α-hydroxy isobutyric acid, D-quinic acid, 2-ethyl-2-hydroxybutyric acid) in aqueous media, led to the successful isolation of binary crystalline Ti(IV)-containing products. The new materials were physicochemically characterized by elemental analysis, FT-IR, TGA, and X-ray crystallography, revealing in all cases the presence of mononuclear Ti(IV) complexes bearing a TiO_6_ core, with three bound ligands of variable deprotonation state. Solution studies through electrospray ionization mass spectrometry (ESI-MS) revealed the nature of species arising upon dissolution of the title compounds in water, thereby formulating a solid-state–solution correlation profile necessary for further employment in biological experiments. The ensuing cytotoxicity profile (pre-adipocytes and osteoblasts) of the new materials supported their use in cell differentiation experiments, thereby unraveling their structure-specific favorable effect toward adipogenesis and mineralization through an arsenal of in vitro biological assays. Collectively, well-defined atoxic binary Ti(IV)-hydroxycaboxylato complexes, bearing bound physiological substrates, emerge as competent inducers of cell differentiation, intimately associated with cell maturation, thereby (a) associating the adipogenic (insulin mimetic properties) and osteogenic potential (mineralization) of titanium and (b) justifying further investigation into the development of a new class of multipotent titanodrugs.

## 1. Introduction

Over the past few decades, extensive interest has focused on the discovery of novel metal complexes with organic substrates as potential alternative choices in the treatment of different types of diseases. Cisplatin was the first metal-based complex, with remarkable anticancer properties, to be used in the treatment of many types of tumors [1,2]. Following that seminal discovery, efforts have been geared toward the design of novel metal-based compounds with anticancer, antimicrobial, and antidiabetic properties [3,4,5]. Among those metal ions, Pt(I,IV), Ru(II,III), Au(I,III), Cu(II), Os(III), and Ti(IV) have been studied mostly due to their special physicochemical characteristics and their favorable biological response [6,7,8]. Titanium is one such transition metal element known for its unique special features. In nature, it is encountered as a component of minerals (rutile, ilmenite, leucoxite) [9]. It is easily processed in the manufacturing industry, playing an important role in the production of many industrial (naval, nuclear power energy, aeronautical industry) as well as consumer products (sunscreens, cosmetics, etc., in the form of white pigments, TiO_2_) [10]. Among its plethoric applications, titanium is considered to be a metal of choice in the manufacturing of implant devices, replacing failed hard and soft tissues. Knee and hip joints and bone plates are some of the hard tissues where titanium is used. Moreover, pure titanium and its alloys are used successfully in dentistry, as components in dental products (bridges, crowns dentures, etc.) [11,12,13]. The excellent biocompatibility that titanium exhibits in combination with its corrosion resistance, balance in its mechanical properties, lightness, low allergic profile, and favorable biological response renders titanium a metal of great importance in the medical field as well [6,8,14]. To that end, titanium has been the subject of research targeting promising anticancer agents with fewer severe side effects and a broad activity spectrum [15,16]. Budotitane (bzac)_2_Ti(OEt)_2_ and titanocene dichloride (Cp_2_TiCl_2_) were the first of such titanium complexes to show promise and were ushered into clinical trials, showing broad anticancer activity and milder side effects in contrast to cisplatin [17,18,19,20,21]. However, formulation issues, fast hydrolysis of titanium compounds toward irreversible formation of inert TiO_2_, and loss of their labile groups in an aqueous solution under physiological conditions emerged as limitations in the clinical trials [5,22,23].

Along the same lines, a potential field in medicinal research is the discovery of novel compounds with anticancer and antidiabetic properties. Diabetes mellitus (with all of its subclassifications, including type 1 and type 2) is a serious metabolic disorder [24]. Hyperglycemia, lipids, carbohydrates, and protein metabolic disorders are the main characteristics of the specific pathology. The increasing appearance in the human population worldwide, combined with the severity of the disease, necessitated the discovery of new suitable drugs over the past few decades. Vanadium (V) is a transition metal located adjacent to titanium (Ti) in the periodic table. The antidiabetic role of vanadium was known over a century ago, with a significant step having taken place in 1985, during an experiment involving the treatment of diabetic rats, induced by streptozotocin, with sodium orthovanadate in drinking water [25]. Many studies have since focused on potential vanadium drugs with antidiabetic properties. Based on the similarities that Ti(IV) and V(V) ions share (similar electronic configuration, structural similarities of titanium and vanadium complexes, etc.) [26,27] and our intense interest in finding novel metal complexes with antidiabetic properties, we were prompted to pursue synthetically and investigate biologically new soluble complex forms of Ti(IV) with low-molecular-mass (α-hydroxycarboxylic acids) ligands, which were ultimately isolated and fully physicochemically characterized. In this research framework, α-hydroxycarboxylic acids belong to a class of substrates of natural origin (Krebs cycle, synthesis of aromatic amino acids, etc.), acting as efficient metal ion chelators bearing two distinctly differentiated coordination moieties, i.e., the α-alcoholic and carboxylic groups, collectively leading to the formation of stable isolable metal ionic complexes [27,28,29,30,31]. To that end, the herein undertaken pH-dependent synthetic effort on Ti(IV)-(α-hydroxycarboxylic acid, L) (L = α-hydroxy isobutyric acid (HIBAH_2_), D-quinic acid (QA), 2-ethyl-2-hydroxy-butyric acid (EHBAH_2_)) systems led to the isolation of four well-defined soluble complex forms. The ensuing biological investigation on those species involved cytotoxic studies, naturally leading to the perusal of their adipogenic and osteogenic potential in different cell lines. This approach provides a well-formulated biological profile of the influence of titanium on adipogenicity and osteogenicity. The employed biological model projected for the first time the bifunctional biological profile, in terms of adipogenic and osteogenic potential, of soluble–bioavailable titanium complex forms with biological substrates, thereby introducing new atoxic forms of an early transition metal ion as viable biomimetic agents linked to metabolism-related (patho)physiology.

## 2. Results

### 2.1. Synthesis

All experimental synthetic procedures between TiCl_4_ and the selected α-hydroxycarboxylic acids took place with simple reagents in H_2_O, under pH-specific conditions. In the case of **1,** TiCl_4_ and α-hydroxy isobutyric acid (HIBAH_2_) reacted in water (molar ratio 1:3) upon addition of aqueous ammonia (1:1) at pH 5. Slow evaporation of the resulting reaction mixture at room temperature led, after a few weeks, to the formation and isolation of colorless crystalline compound **1**, in line with the stoichiometric Reaction (1) that follows.

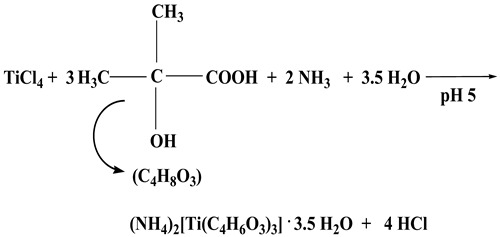
(1)

Through a similar reaction, TiCl_4_ and α-hydroxy isobutyric acid (HIBAH_2_) (molar ratio 1:3) reacted in the presence of guanidinium carbonate, in an aqueous solution with pH 6.5–7.0. A subsequent slow evaporation process at room temperature resulted in the isolation of compound **2** (Reaction (2)).
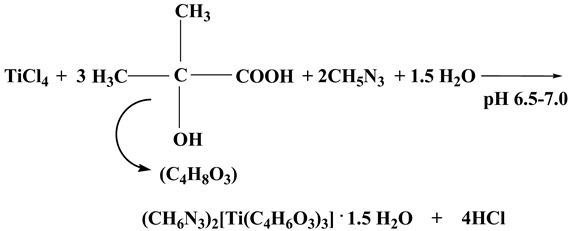
(2)

The synthesis and isolation of compound **3** were the result of the reaction between TiCl_4_ and D-quinic acid (1:2 experimental molar ratio) in an aqueous solution, with the addition of an aqueous solution of ammonia (1:1) affording a final pH value of 6.5. The addition of ethanol at 4 °C afforded crystalline material **3**. The stoichiometric synthesis of compound **3** is shown below, according to Reaction (3).
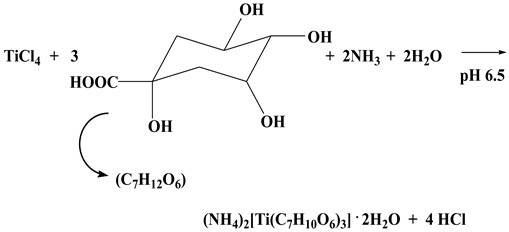
(3)

Finally, in the synthesis of compound **4**, TiCl_4_ reacted with the selected ligand 2-ethyl-2-hydroxybutyric acid in water (experimental molar ratio 1:3). The addition of an aqueous solution of ammonia (1:1), in order to adjust the pH of the reaction to a final value of 5, was crucial for the isolation of the specific complex. The resulting reaction mixture was allowed to stand at room temperature and slowly evaporate. The overall stoichiometric Reaction (4) is presented below.
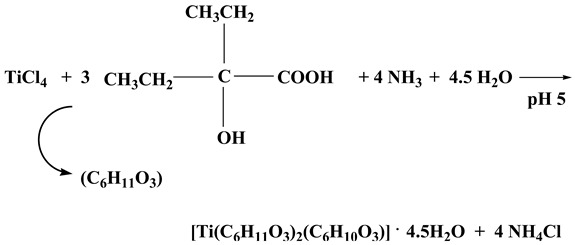
(4)

Compounds **1**–**4** are stable in the crystalline form, at room temperature, in the air. Elemental analysis suggested the formation of (NH_4_)_2_[Ti(C_4_H_6_O_3_)_3_]∙3.5H_2_O (**1**), (CH_6_N_3_)_2_[Ti(C_4_H_6_O_3_)_3_]∙1.5H_2_O (**2**), (NH_4_)_2_[Ti(C_7_H_10_O_6_)_3_]∙2H_2_O (**3**), and Ti(C_6_H_11_O_3_)_2_(C_6_H_10_O_3_)·4.5H_2_O (**4**) compounds. Further, FT-IR spectroscopy, and X-ray crystallography positively identified all compounds **1**–**4**. All compounds are soluble in H_2_O, a prerequisite for further biological investigation.

### 2.2. Description of X-ray Crystallographic Structures

The X-ray crystal structure determination of the new materials (Table 1) revealed that compounds **1**–**3** consist of discrete anionic and cationic assemblies, except for compound **4**, which consists of neutral molecules. In **1**–**3**, the cations arising through base addition (aqueous ammonia and/or aqueous solution of guanidinium carbonate), for pH adjustment of the reaction mixture, act as counterions, thus contributing to the formation and stability of their final solid-state architecture. In all cases, the α-hydroxycarboxylic acids act as bidentate chelating ligands, coordinating to Ti(IV) ions through the carboxylato group and the α-alcoholato oxygen anchors. All carboxylic acid groups of all participating ligands in the coordination sphere of Ti(IV) were found to be deprotonated. Furthermore, the α-alcoholic groups of the ligands **1**, **2**, and **3** were found to be deprotonated. In the case of compound **4**, the same anchor groups were either protonated and/or deprotonated.

**Figure 1:**
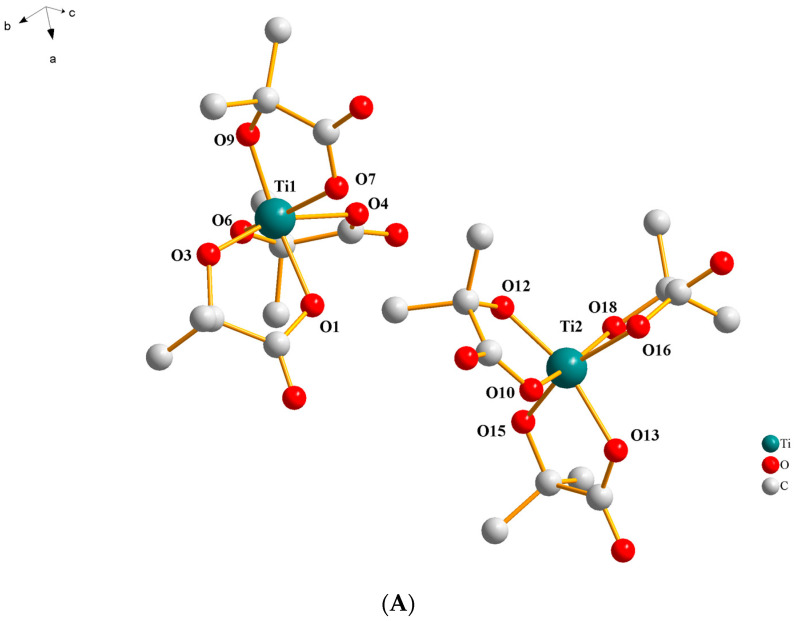

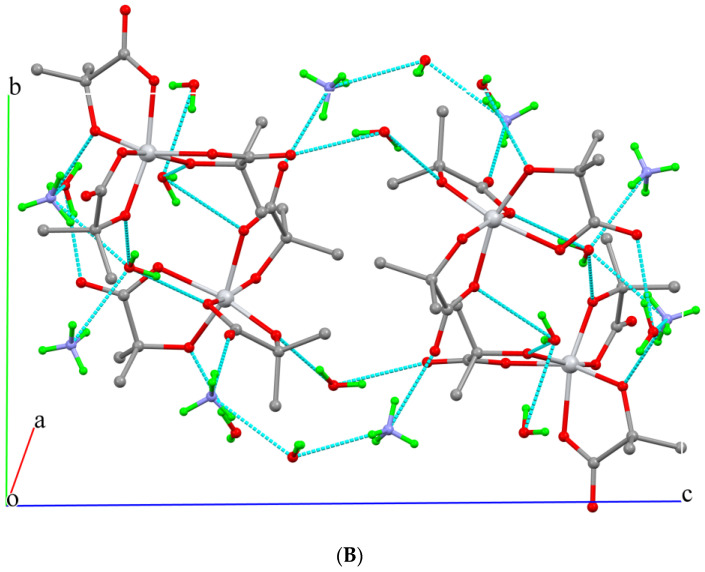
(**A**) Diamond plot of the anionic assembly [Ti(C_4_H_6_O_3_)_3_]^2−^ in (NH_4_)_2_[Ti(C_4_H_6_O_3_)_3_]·3.5H_2_O (**1**). (**B**) Hydrogen-bonding interactions in **1** (cyan dotted lines). Hydrogen atoms are provided in green color. Oxygen atoms are provided in red color. Nitrogen atoms are provided in blue color.

More specifically, compound **1** crystallizes in the triclinic space group *P*ī, with the asymmetric unit assembled from two slightly different Ti(IV) complexes. In addition, four ammonium countercations and seven solvent water molecules complete the structure’s architectural contents. In the coordination sphere of Ti(IV), there exist three α-hydroxy isobutyrato (−2) doubly deprotonated ligands bound to the metal ion in a bidentate fashion through one carboxylato and the α-alcoholato oxygen atoms (Figure 1A). The molecular structure of the complex species [Ti(C_4_H_6_O_3_)_3_]^2−^ reveals that the geometry around Ti(IV) is highly distorted octahedral, with the most axial vector being 160.26(5)° among O(1), Ti(1), and O(9) and 161.65(6)° among O(15), Ti(2), and O(16). The generated bite angles are quite similar, varying between 78.95(6)° and 79.33(6)° in the first complex assembly and 78.46(6)° and 79.81(7)° in the second complex assembly present in the asymmetric unit. The Ti(IV) to α-alcoholato oxygen atom distances vary from 1.8666(15) to 1.8742(15) Å, being shorter than the distances to the carboxylato oxygen atoms, which vary from 2.0363(17) to 2.0644(16) Å. Selected bond lengths and angles of the complex assemblies are presented in Table 2.

Ammonium counterions neutralize the charge from the dianionic complex assemblies, with solvent water molecules filling the lattice voids formed. One of the water molecules was found to be disordered over three positions, with occupancy factors ½, ¼, and ¼. A hydrogen-bonding net forms from all of these participating molecules and ions. All hydrogen atoms connected to oxygen and most of them connected to nitrogen atoms participate in a network of thirty-four interactions, forming a rigid 3D lattice, as shown in Figure 1B.

**Figure 2:**
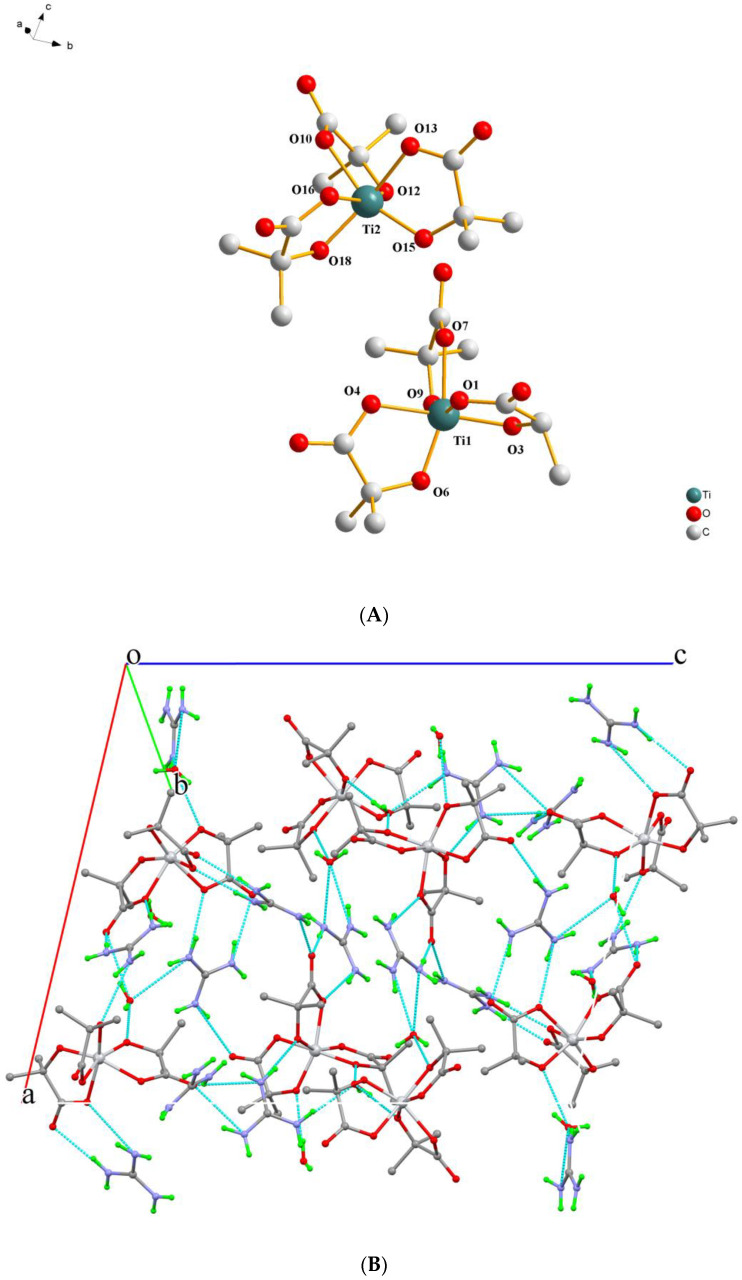
(**A**) Diamond plot of the anionic assembly [Ti(C_4_H_6_O_3_)_3_]^2−^ in (CH_6_N_3_)_2_[Ti(C_4_H_6_O_3_)_3_]∙1.5H_2_O (**2**). (**B**) Hydrogen-bonding interactions in **2** (cyan dotted lines). Hydrogen atoms are provided in green color. Oxygen atoms are provided in red color. Nitrogen atoms are provided in blue color.

Compound **2** crystallizes in the monoclinic space group *P*2_1_*/n.* The asymmetric unit contains two Ti(IV)-(α-hydroxy isobutyrato) complexes, four guanidinium counterions, and three solvent water molecules, all located in general positions. The geometry of the Ti(IV) complexes [Ti(C_4_H_6_O_3_)_3_]^2−^ is quite similar to that found in the corresponding assemblies in **1**. The metal ion is six-coordinate, and the doubly deprotonated ligands are coordinated in the same mode, bearing similar geometric parameters (distances and angles) with respect to the center (Figure 2A). Selected bond lengths and angles of the complexes are presented in Table 2.

Hydrogen atoms from the lattice water molecules and the guanidinium counterions interact among them and with the oxygen atoms of the α-hydroxybutyrato ligands, thus resulting in a hydrogen-bonding network. These twenty-nine interactions extend in all directions in the crystal lattice, bridging the molecules and the ions together and giving rise to a rigid 3D crystal lattice, as shown in Figure 2B.

**Figure 3:**
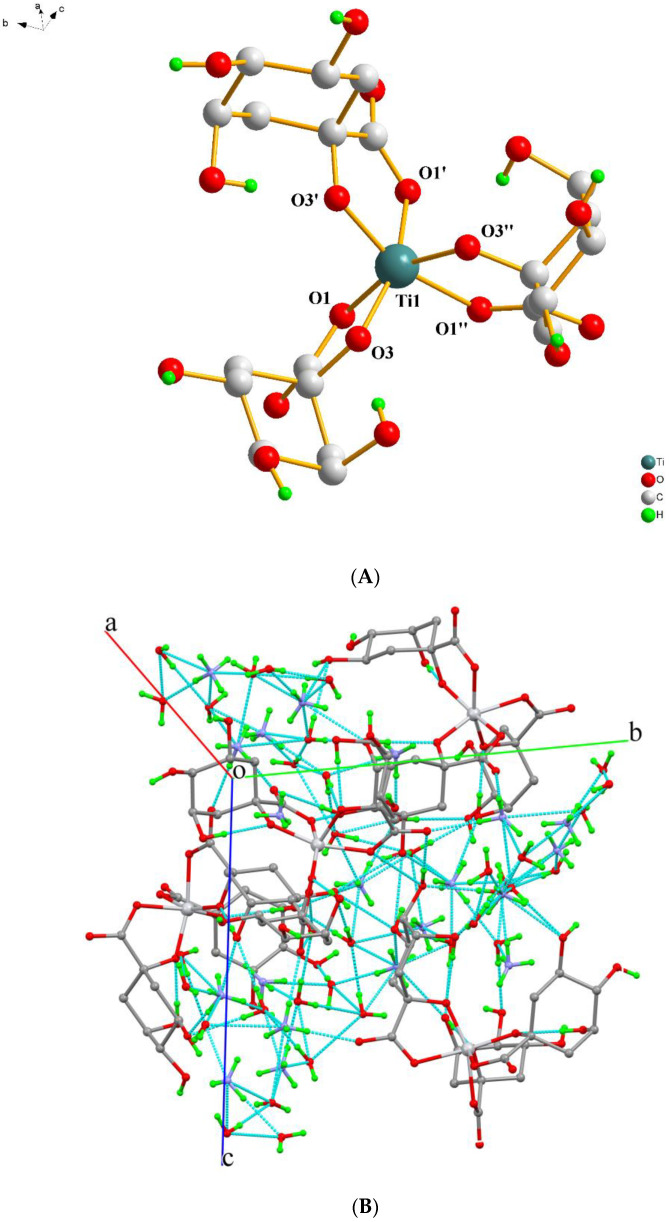
(**A**) Diamond plot of the anionic assembly [Ti(C_7_H_10_O_6_)_3_]^2−^ in (NH_4_)_2_[Ti(C_7_H_10_O_6_)_3_]·2H_2_O (**3**). (**B**) Hydrogen-bonding interactions in **3** (cyan dotted lines). Hydrogen atoms are provided in green color. Oxygen atoms are provided in red color. Nitrogen atoms are provided in blue color.

Compound **3** crystallizes in the non-centrosymmetric cubic space group *P*2_1_3. The unit cell contains a total of four Ti(IV) complexes, eight ammonium counterions, and eight lattice water molecules. The asymmetric unit contains one Ti(IV) metal ion, located in a special position, with a multiplicity of three and full occupancy. To that center, one doubly deprotonated (−2) quinic acid ligand is bound through the carboxylato and α-alcoholato group oxygen anchors. The remaining alcoholic groups of the ligand remain protonated. All of the atoms of the ligand are located in general positions and multiplied by 3 due to symmetry considerations. The bond distances around the Ti(IV) center (Ti(1)—O(1) 2.039(3) and Ti(1)—O(3) 1.866(2) Å) and the bite angle O(1)—Ti(1)—O(3) with the value of 78.57(11)° are typical for this type of complex [32]. The geometry around Ti(IV) is distorted octahedral. The structure of the complex [Ti(C_7_H_10_O_6_)_3_]^2−^ is presented in Figure 3A. The carbon skeleton of the quinato ligand adopts the chair conformation, with the carbon atoms C(4) and C(6) being chiral and adopting the *R* conformation. The absolute structure assignment is also consistent with the lowest value of the Flack parameter, −0.02(6), resulting from 1067 Friedel pairs.

The eight ammonium counterions of the unit cell are symmetrically generated from three ammonium ions, placed in special and general positions, with different occupancy factors. The eight lattice water molecules, on the other hand, are generated from three water molecules sitting in general positions. All hydrogen atoms of the lattice water molecules, the ammonium counterions, and the protonated alcoholic groups of the ligands interact together with oxygen and nitrogen atoms from the same groups, thus creating an extended hydrogen-bonding network. These twenty-one interactions form a rigid 3D crystal lattice, as shown in Figure 3B.

**Figure 4:**
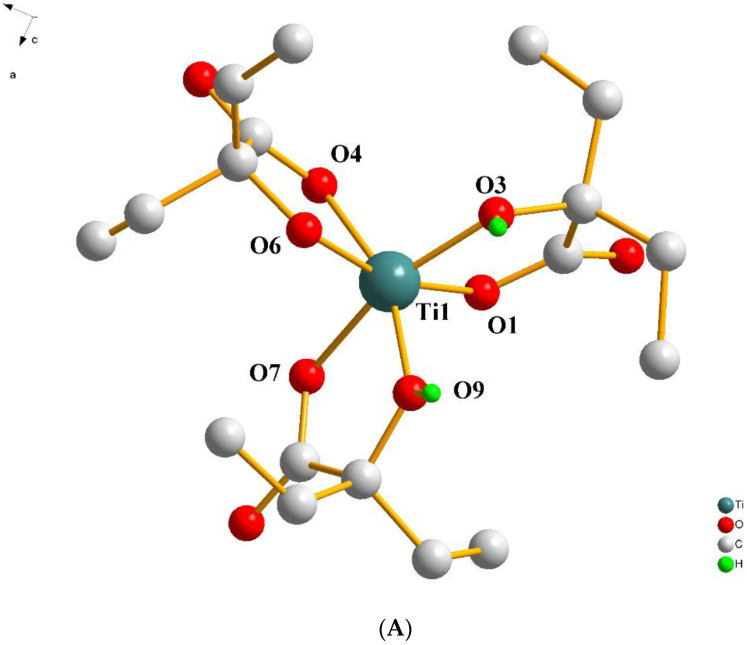

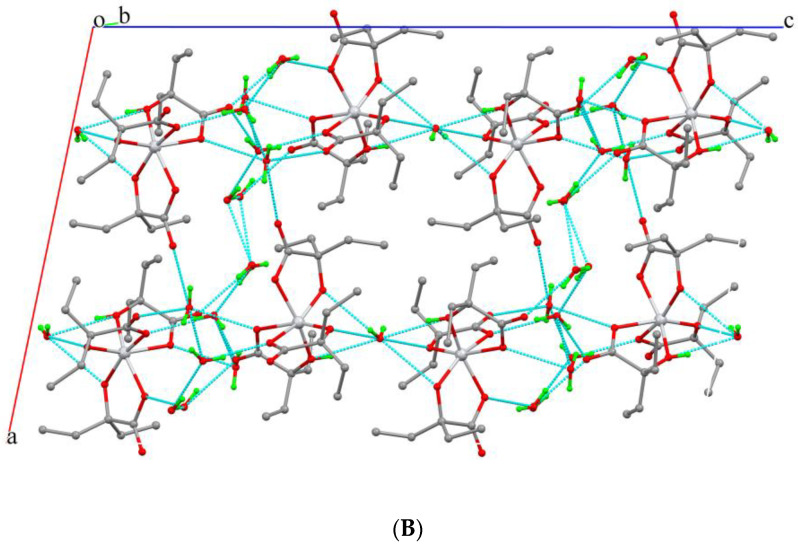
(**A**) Diamond plot of the complex assembly [Ti(C_6_H_11_O_3_)_2_(C_6_H_10_O_3_)] in [Ti(C_6_H_11_O_3_)_2_(C_6_H_10_O_3_)]·4.5H_2_O (**4**). (**B**) Hydrogen-bonding interactions in **4** (cyan dotted lines). Hydrogen atoms are provided in green color. Oxygen atoms are provided in red color.

Complex **4** crystallizes in the monoclinic space group *C*2/*c*. The unit cell contains eight neutral Ti(IV) complexes and thirty-six lattice water molecules. Two of the 2-ethyl-2-hydroxybutyric acid ligands were found to be singly deprotonated (only in the carboxylic acid group). The third one was found to be doubly deprotonated in the carboxylic acid and the α-alcoholic groups. All ligands act as chelate bidentate agents, coordinated to the metal ion through one carboxylato and the α-alcoholato(ic) oxygen atoms. The geometry around the metal ion is distorted octahedral, with the carboxylato oxygen atoms O(1), O(4), and O(7) being placed at longer distances than the alcoholato(ic) oxygen atoms O(3), O(6), and O(9). The bite angles generated from the pairs O(1) and O(3), O(4) and O(6), and O(7) and O(9), having the Ti(IV) center as a pivot atom, are very similar (77.99(6), 78.66(7), and 78.31(7)°, respectively). The structure of the complex assembly [Ti(C_6_H_11_O_3_)_2_(C_6_H_10_O_3_)] is presented in Figure 4A. The carbon atoms in two of the three ligands have been found to be disordered. In two of the ligands, one terminal methyl group has been found disordered over two positions with equal occupancy factors. Moreover, in one of these ligands, the ethyl group was also found disordered over two positions, with equal occupancy factors for the disordered atoms.

All hydrogen atoms from the protonated alcoholic groups and the solvent water molecules participate in the assembly of a hydrogen-bonding interaction network, keeping the molecules close together and forming a rigid 3D lattice, as shown in Figure 4B.

The Ti-O bond lengths in **1**–**4** are in the range 1.827(2)–2.072(2) Å, being in line with those in similar Ti(IV)-O_6_ complexes, such as K_3_[Ti(C_6_H_6_O_7_)_2_(C_6_H_5_O_7_)]·K_4_[Ti(C_6_H_5_O_7_)_2_(C_6_H_6_O_7_)]·10H_2_O (1.860(2)–2.060(3) Å) (**5**), (C_14_H_13_N_2_)_2_[Ti(C_6_H_6_O_7_)_3_]·5H_2_O (1.875(2)–2.014(2) Å) (**6**), Na_3_[Ti(C_6_H_6_O_7_)_2_(C_6_H_5_O_7_)]·9H_2_O (1.861(2)–2.064(2) Å) (**7**) [30], K_4_[Ti(C_6_H_6_O_7_)(C_6_H_5_O_7_)_2_]·4H_2_O (1.850(3)–2.069(3) Å) (**8**), K_5_[Ti(C_6_H_5_O_7_)_3_]·4H_2_O (1.843(5)–2.067(5) Å) (**9**) [33], and (NH_4_)_4_[Ti_2_(O_2_)_2_(C_6_H_4_O_7_)_2_]·2H_2_O (1.852(2)–2.085(2) Å) (**10**) [34].

### 2.3. FT-IR Spectroscopy

The FT-infrared spectra of **1**–**4** in KBr revealed vibrationally active carboxylato groups belonging to the deprotonated α-hydroxycarboxylic acid groups bound to the metal ion (Ti(IV)). Antisymmetric and symmetric vibrations for the metal-bound carboxylato moieties were present in all recorded spectra. Specifically, antisymmetric stretching vibrations v_as_(COO^–^) for the carboxylate carbonyls emerged at 1632, 1680, 1650, and 1628 cm^−1^ for compounds **1**–**4**, respectively, whereas the symmetric vibrations v_s_(COO^–^) for the same groups appeared in the range 1461–1327 for **1**, 1473–1327 for **2**, 1468–1327 for **3**, and 1460–1327 cm^−1^ for **4**. In all cases, the observed carbonyl vibrations were shifted to lower frequency values in comparison to the corresponding vibrations in the free ligands. The observation suggests changes in the vibrational status of the α-hydroxy-isobutyrato ([HIBA]^2−^), D-quinato, and 2-ethyl-2-hydroxy-butyrato ligands upon coordination to the Ti(IV) ion.

### 2.4. Thermal Studies

**Figure 5:**
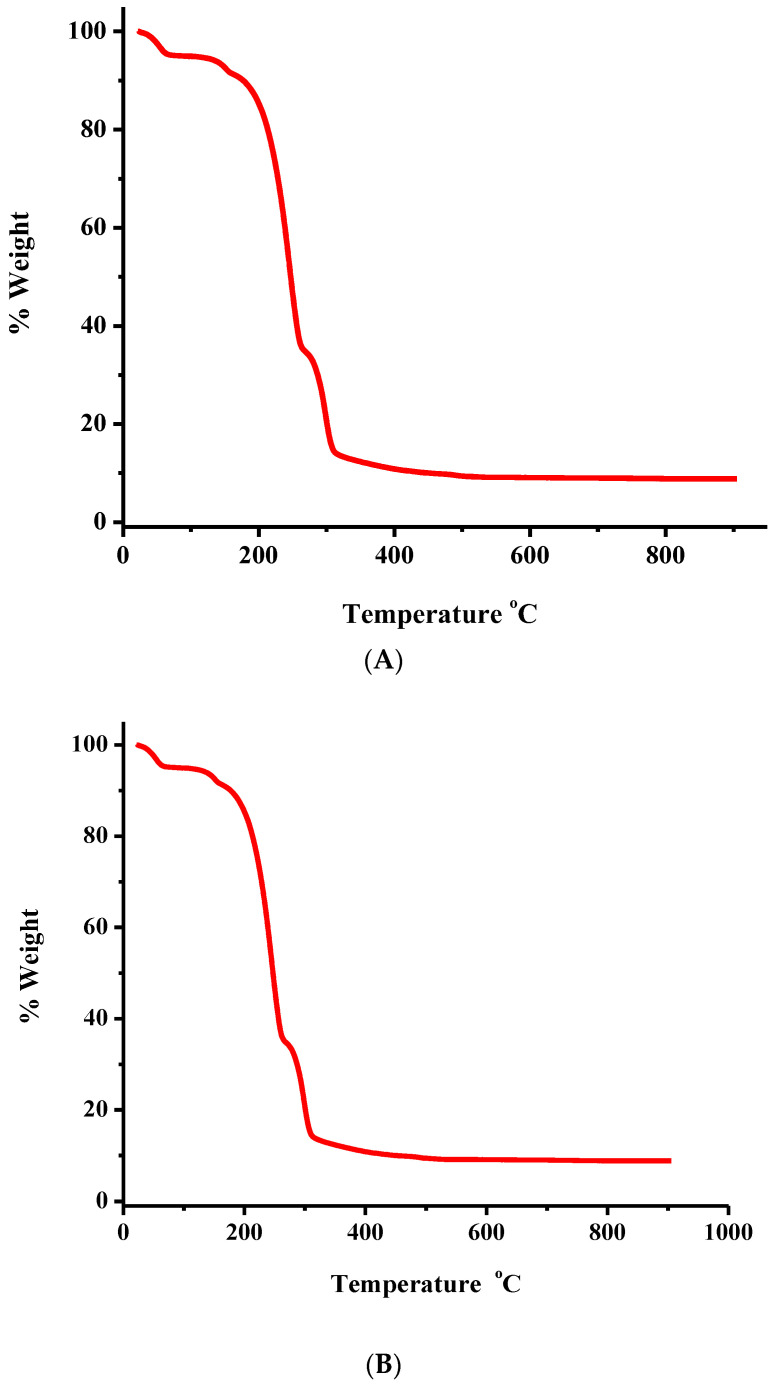
TGA plots of compounds **1** (**A**), and **2** (**B**).

The thermal decomposition of **1** and **2** was studied, as a representative token of the family of derived species, by thermogravimetric analysis (TGA) under an oxygen atmosphere (Figure 5A,B). Complex **1** is stable up to 30 °C. Beyond that point, up to 505 °C, a continuous mass loss is observed, initially reflecting the removal of lattice water and after that reflecting the decomposition of the organic part of the compound (Figure 5A). As observed during these stages, there are no clear plateaus exhibiting no mass change. This suggests that the arising intermediates are unstable, undergoing further decomposition. Beyond the temperature of 505 °C, no further decomposition is observed, consistent with TiO_2_ being the final solid-state product for **1** and CO_2_, H_2_O, and N_2_ representing generated products released in the gas state.

Similarly, compound **2** has its own unique profile (Figure 5B). Specifically, from 30 °C and up to 490 °C, mass loss occurs as a result of lattice water removal and further decomposition of the organic matrix of the compound. Throughout these stages, no clear plateaus are observed, suggesting instability and further decomposition of the derived products. From 490 °C to 900 °C, the mass remains stable, indicating that the final decomposition solid-state product is the TiO_2_, with CO_2_, H_2_O, and N_2_ representing generated products released in the gas state.

### 2.5. Mass Spectrometry Measurements

Mass spectrometry measurements of **1**–**3** reveal their identity in an aqueous solution, with the ESI-MS measurements run, upon dissolution of the respective compounds, in positive mode. For **1**, M_1_ = [Ti(L_1_)_3_] (*m*/*z* = 357.0683, z = 1, [M_1_H_3_]^+^) (Figure S1), for **2**, M_2_ = [Ti(L_1_)_3_((CH_6_N_3_)_2_)] (*m*/*z* = 475.1627, z = 1, [M_2_H]^+^) (Figure S2), for **3**, M_3_1_ = [Ti(L_2_)_3_] (*m*/*z* = 621.1262, z = 1, [M_3_1_H_3_]^+^) and M_3_2_ = [Ti(L_2_)_2_] (*m*/*z* = 429.0590, z = 1, [M_3_2_H]^+^) (Figure S3), and for **4**, M_4_ = [Ti(L_3_H)_2_(L_3_)] (*m*/*z* = 479.1509, z = 1, [M_4_K]^+^) (Figure S4).

### 2.6. Cytotoxicity Studies

**Figure 6:**
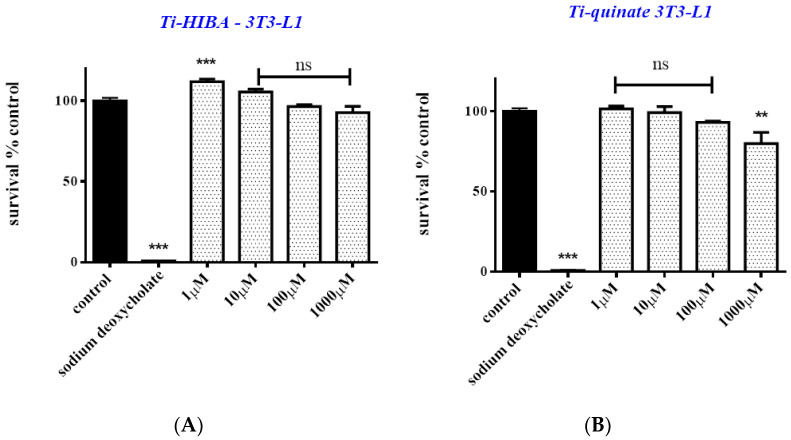

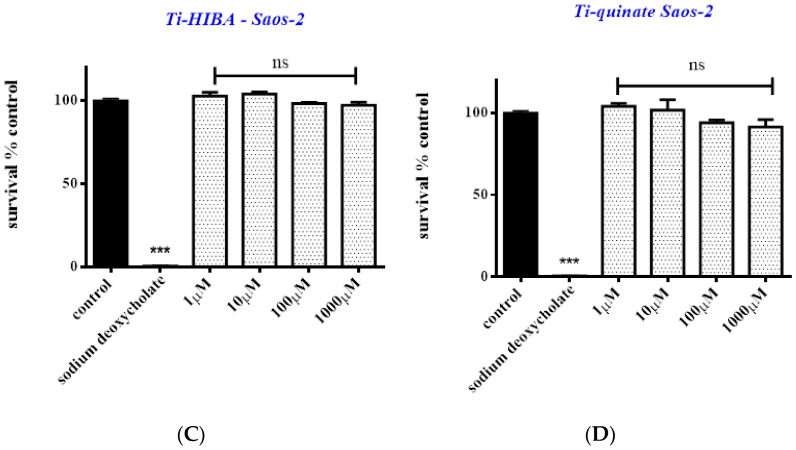
Percent change of cell survival in 3T3-L1 pre-adipocytes (**A**,**B**) and Saos-2 osteoblasts (**C**,**D**), following treatment with various concentrations (1–1000 μM) of Ti(IV)-HIBA (**1**) and Ti(IV)-quinic (**3**) for 24 h. Sodium deoxycholate was used as a positive control. Values represent the mean value of several (n = 3) independent experiments. Vertical bars represent SEMs. ** *p* < 0.01 (highly significant), *** *p* < 0.001 (extremely significant) or non-significant (*p* > 0.05) vs. control.

Prior to investigating the biomimetic activity of the employed compounds, the (a)toxic profile of the new titanium species was investigated. Both pre-adipocytes (3T3-L1) and osteoblasts (Saos-2) were exposed to **1** and **3**, for 24 and 48 h, at various concentrations ranging from 1 μΜ to 1000 μΜ. As shown in Figure 6, both complexes appear to be non-toxic when cells are treated over short-term incubation periods, both in the case of 3T3-L1 and Saos-2 cell lines. More specifically, cell survival in 3T3-L1 cultures over 24 h amounts to 112.0% (*p* < 0.001), 105.6% (*p* > 0.05), 96.5% (*p* > 0.05), and 92.7% (*p* > 0.05) when cells are treated with 1, 10, 100, and 1000 μΜ of **1** (Figure 6A), and 101.6% (*p* > 0.05), 99.3% (*p* > 0.05), 93.2% (*p* > 0.05), and 80% (*p* < 0.01) when cells are treated with 1, 10, 100, and 1000 μΜ of **3** (Figure 6B), respectively. Cell survival is not affected in the case of Saos-2 either. Cell survival, for the same period of exposure and concentration range, amounts to 102.7%, 104.0%, 98.2%, and 97.3% (*p* > 0.05) in the case of **1** (Figure 6C) and 104.0%, 101.8%, 94.0%, and 91.5% (*p* > 0.05) in the case of **3** (Figure 6D).

**Figure 7:**
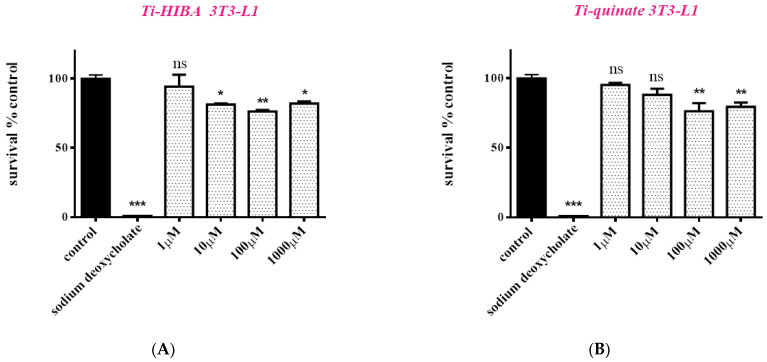

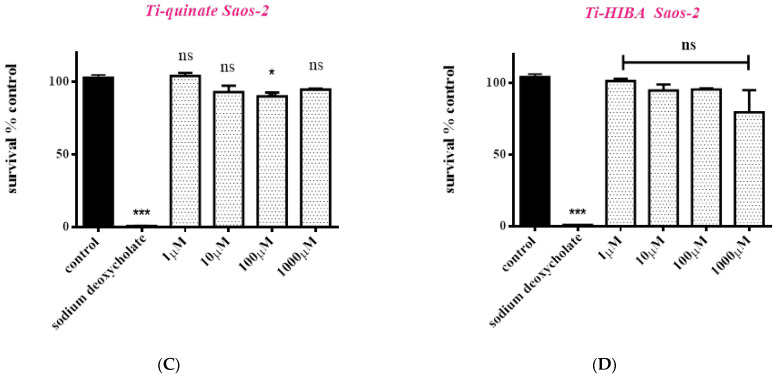
Percent change of cell survival in 3T3-L1 pre-adipocytes (**A**,**B**) and Saos-2 osteoblasts (**C**,**D**), following treatment with various concentrations (1–1000 μM) of Ti(IV)-HIBA (**1**) and Ti(IV)-quinic (**3**) for 48 h. Sodium deoxycholate was used as a positive control. Values represent the mean value of several (n = 3) independent experiments. Vertical bars represent SEMs. * *p* < 0.05 (significant), ** *p* < 0.01 (highly significant), *** *p* < 0.001 (extremely significant) or non-significant (*p* > 0.05) vs. control.

By the same token, cells were also exposed to **1** and **3** for 48 h (Figure 7). In the case of 3T3-L1 cell cultures, when cells are treated with **1**, cell survival amounts to 94.2% (*p* > 0.05), 81.4% (*p* < 0.05), 76.4% (*p* < 0.01), and 82.0% (*p* < 0.05) for 1, 10, 100, and 1000 μΜ, respectively (Figure 7A). When cells are treated with **3**, cell survival amounts to 95.3% (*p* > 0.05), 88.2% (*p* > 0.05), 76.3% (*p* < 0.01), and 79.4% (*p* < 0.01) for 1, 10, 100, and 1000 μΜ, respectively (Figure 7B). Similarly, in the case of Saos-2 cultures, when cells are treated with **1** in the same concentration range, cells survival amounts to 104.1% (*p* > 0.05), 93.0% (*p* > 0.05), 89.9% (*p* < 0.05), and 94.6% (*p* > 0.05) for 1, 10, 100, and 1000 μΜ, respectively (Figure 7C). In the case of **3**, cell survival amounts to 101.4%, 94.8%, 95.5%, and 79.6% (*p* > 0.05) for 1, 10, 100, and 1000 μΜ, respectively (Figure 7D). Taken together, the results show that both complexes can be used for further biomimetic assays for concentrations up to 100 μΜ.

### 2.7. Cell Migration

**Figure 8:**
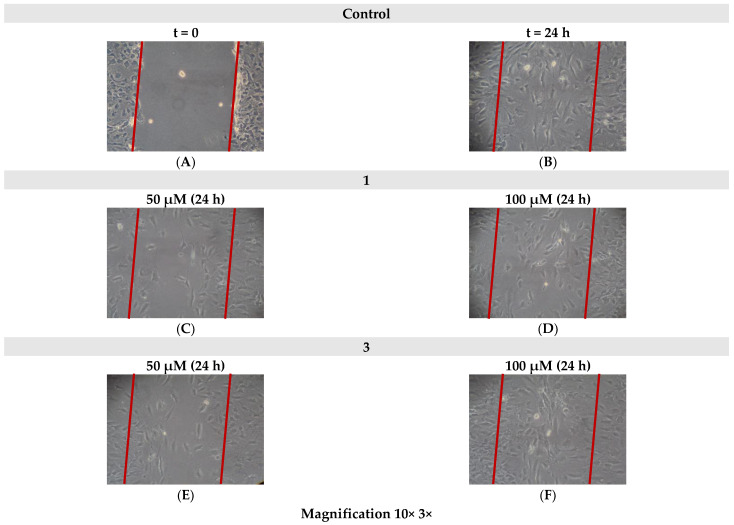
Cell migration of 3T3-L1 (**A**) control (t = 0), (**B**) control after 24 h, (**C**) cells treated with 50 μΜ of **1**, (**D**) 100 μΜ of **1**, (**E**) cells treated with 50 μΜ of **3**, and (**F**) 100 μΜ of **3**, after 24 h, using an in vitro standard scratch assay.

In an effort to investigate the potential effects of the title compounds on the endogenous cell migration ability of 3T3-L1 pre-adipocytes, an in vitro scratch assay was performed. The physiological migrating activity of cells, grown in the presence of only DMEM, was considered as a control. Cells were treated with either 50 μΜ or 100 μΜ of each Ti(IV)-compound for 24 h. Monitoring of the cell migratory progress was pursued through microscopic examination of samples (10× 5× magnification). As shown in Figure 8, cells exhibited normal migration in the case of both Ti(IV)-compounds after 24 h at 100 μΜ compared to the control (untreated cells). Worth mentioning is the fact that in the case of the 50 μΜ concentration of Ti(IV)-compound samples, a slight reduction in cell motility was observed. With respect to cell morphology, no aberration from the normal state of both cases of employed Ti(IV)-compounds was observed.

### 2.8. Study of In Vitro Adipogenesis

**Figure 9:**
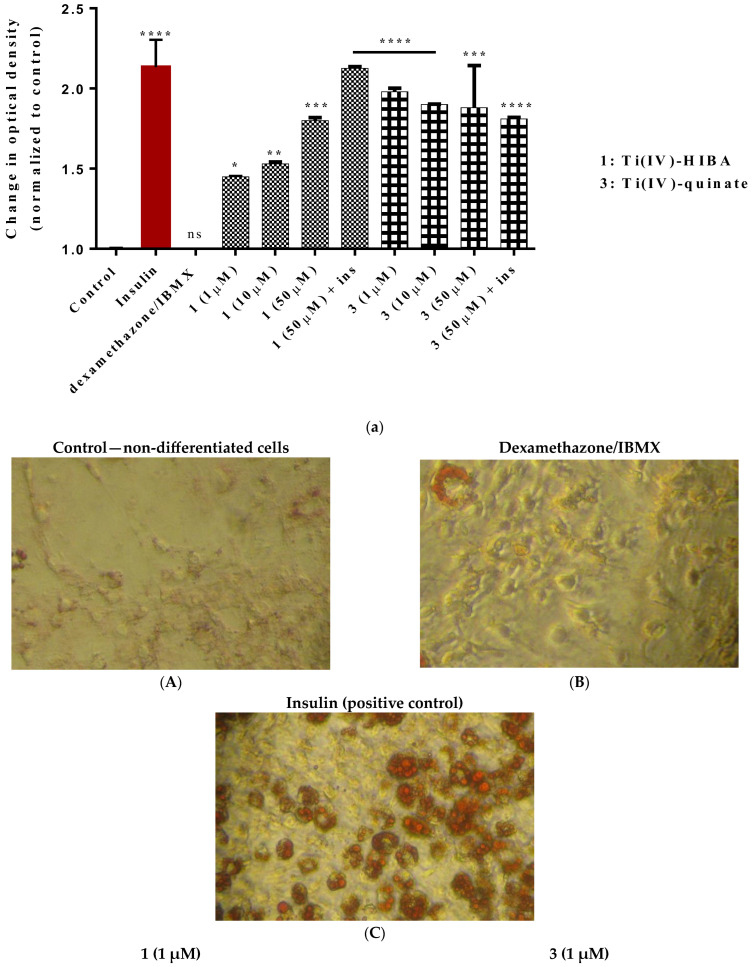

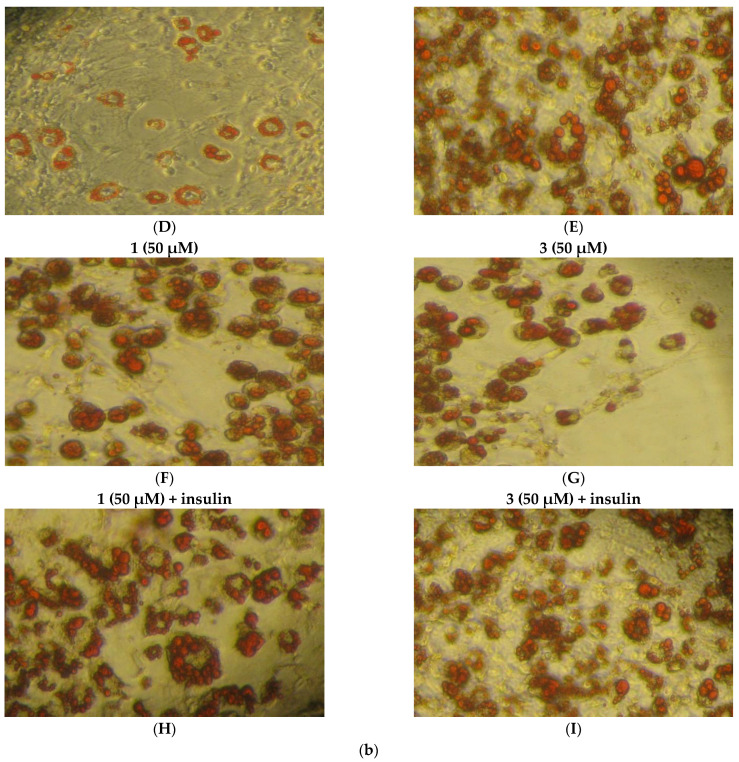
(**a**) Relative change in optical density of **1** and **3** after adipogenesis, compared to pre-adipocytes (control, untreated cells), in 3T3-L1 cell cultures. Values represent the mean value of several (n = 3) independent experiments. Vertical bars represent SEMs. * *p* < 0.05 (significant), ** *p* < 0.01 (highly significant), *** *p* < 0.001 (extremely significant), **** *p* ≤ 0.0001 (extremely significant), or non-significant (*p* > 0.05) vs. control. (**b**) Representative micrographs of 3T3-L1 pre-adipocyte differentiation, assessed by oil red O staining. Images were captured using an AxioCamHc camera with a 40× phase contrast lens. (**A**) Untreated cells, (**B**) adipocytes differentiated in the presence of dexamethazone/IBMX, (**C**) adipocytes differentiated in the presence of insulin (10 ng/mL), (**D**) **1** (1 μΜ), (**E**) **3** (1 μΜ), (**F**) **1** (50 μΜ), (**G**) **3** (50 μΜ), (**H**) combination of **1** (50 μΜ) and insulin, and (**I**) combination of **3** (50 μΜ) and insulin.

To investigate the adipogenic potential and thus insulin-mimetic and/or -enhancing activity of Ti(IV)-complexes, the title compounds were employed in experiments targeting induction of cell differentiation of 3T3-L1 pre-adipocytes into mature adipocytes, following a standard differentiation protocol. As shown in Figure 9a, compound **1** appears to induce adipogenesis in a concentration-dependent manner. More specifically, the relative optical density is 1.45 (*p* < 0.05), 1.53 (*p* < 0.01), and 1.80 (*p* < 0.001) when cells are treated with 1, 10, or 50 μΜ of compound **1** (total replacement of insulin), whereas when **1** is used in combination with insulin, the optical density is 2.10 (*p* ≤ 0.0001). In contrast, when cells are treated with **3**, the effect seems to be independent of the concentration or the presence of insulin, since the optical density is ~1.88 (*p* ≤ 0.0001). Representative pictures of the lipid droplets, forming on the 8th day of the differentiation process, are shown in Figure 9b. All of the obtained results were comparable to the differentiation effect induced by insulin (10 ng/mL), which serves as a positive control in the assay.

### 2.9. Study of In Vitro Mineralization

**Figure 10:**
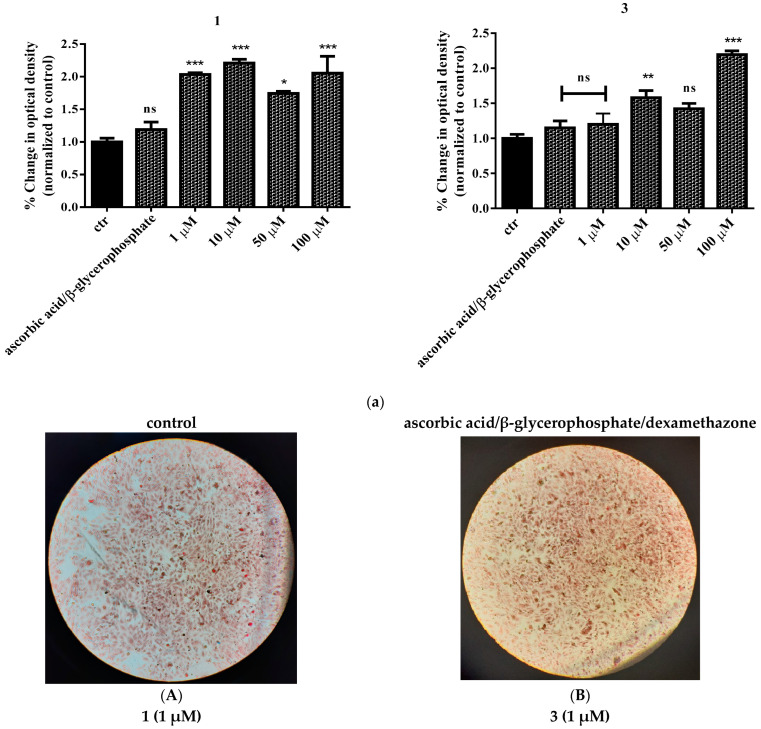

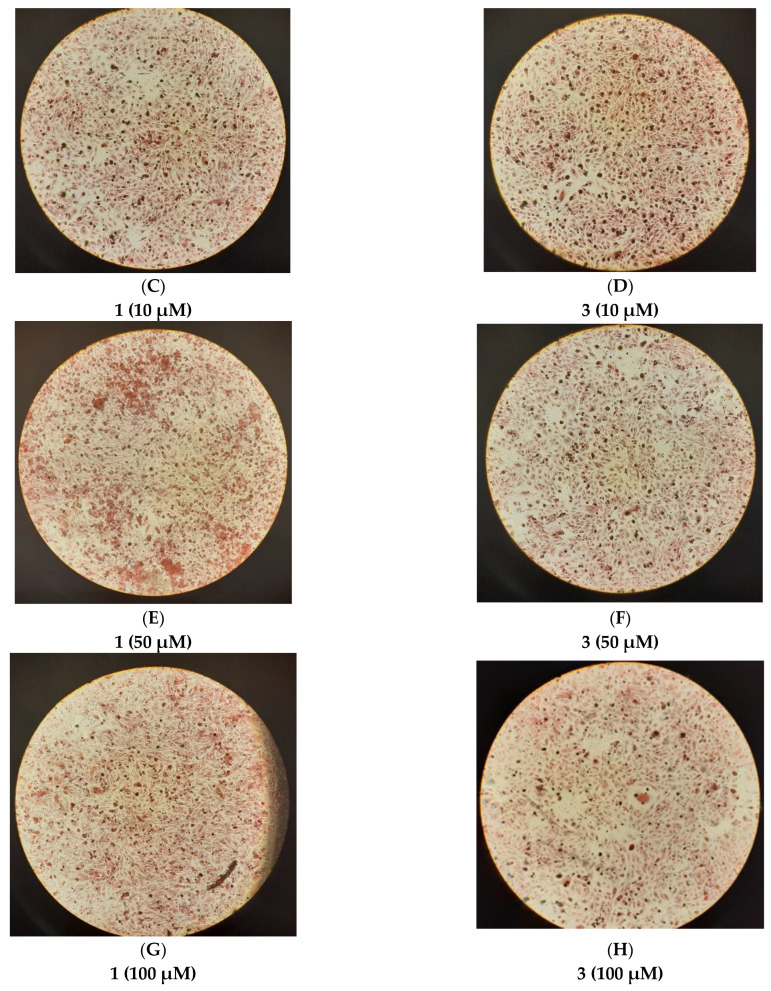

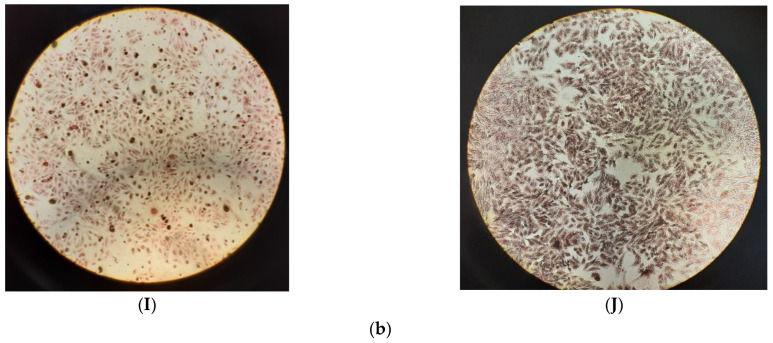
Relative change in optical density of **1** and **3** (**a**) following mineralization, compared to pre-osteoblasts (control, untreated cells), in Saos-2 cell cultures. Values represent the mean value of several (n = 3) independent experiments. Vertical bars represent SEMs. * *p* < 0.05 (significant), ** *p* < 0.01 (highly significant), *** *p* < 0.001 (extremely significant) or non-significant (*p* > 0.05) vs. control. (**b**) Representative micrographs of Saos-2 differentiation, assessed through Alizarin red staining. Images were captured using an AxioCamHc camera with a 10× phase contrast lens. (**A**) Untreated cells, (**B**) cells differentiated in the presence of ascorbic acid and β-glycerophosphate, (**C**) **1** (1 μΜ), (**D**) **3** (1 μΜ), (**E**) **1** (10 μΜ), (**F**) **3** (10 μΜ), (**G**) **3** (50 μΜ), (**H**) **3** (50 μΜ), (**I**) **3** (100 μΜ), and (**J**) **3** (100 μΜ).

**Figure 11:**
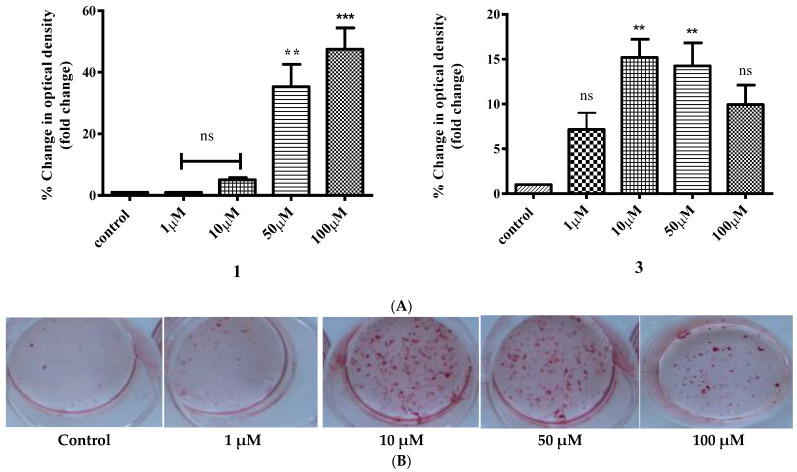
Relative change in optical density of **1** and **3** in KS483 cells (**A**) following mineralization testing in a concentration-dependent manner (1–100 μM), compared to pre-osteoblasts (control, untreated cells). Values represent the mean value of several (n = 3) independent experiments. Vertical bars represent SEMs. ** *p* < 0.01 (highly significant), *** *p* < 0.001 (extremely significant) or non-significant (*p* > 0.05) vs. control. (**B**) Representative pictures of plates showing concentration-dependent (1–100 μM) mineralization activity in KS483 pre-osteoblastic cells for compound **3** in comparison to the control.

In order to examine the effect of the title compounds on the cell mineralization process, Saos-2 cells were treated with various concentrations (1–100 μΜ) of **1** or **3**. A standard mineralization protocol was followed as described below (Section 4). Exposure to ascorbic acid and β-glycerophosphate was used as the positive control in the assay. As shown in Figure 10a, cells were successfully differentiated in all cases tested, and the result was significant compared to the positive control for both complexes. Worth mentioning is the fact that the highest effect observed was achieved at 10 μΜ in the case of **1** and 100 μΜ in the case of **3**, indicating an enhancing effect, since the compounds were used in the presence of the mineralization induction medium. Representative pictures of mineral deposits forming on the 14th day of the differentiation process are shown in Figure 10b. To further investigate the effect of the title compounds on mineralization, KS483 pre-osteoblasts were also employed. The effect of the various compounds on mineralization in KS483 cells is depicted in Figure 11. We herein report a concentration-dependent increase in Alizarin red staining of both Ti(IV) compounds compared to unstimulated cells (Figure 11), reaching up to ~47% in the case of **1** (100 μΜ).

## 3. Discussion

### 3.1. Synthetic Challenges in the Pursuit of Biological Titanium Candidates

Titanium, albeit a transition metal ion plentifully encountered in industrial applications, has been increasingly drawing attention as a biomaterial component and potential metallopharmaceutical in a number of pathophysiological aberrations [35,36,37,38,39]. A serious impediment to such an effort is its natural propensity to drive aqueous reactivity toward the ultimate product TiO_2_. Its hydrolytic instability in aqueous media, combined with potential chelate chemical reactivity with hydroxycarboxylic acid ligands stabilizing Ti(IV), stands as a major challenge in the generation of Ti(IV)-ligand species capable of exerting biological influence in a comprehensive and atoxic fashion. Over the years, several attempts were made to synthesize complexes of Ti(IV), with the prospect of using them as therapeutic drugs (e.g., in cancer treatment) [35,36,37]. In meeting the great challenge of synthesizing well-defined soluble and bioavailable complex species of Ti(IV) (a) bearing physiological substrates as ligands, and (b) being capable of exerting biological effects linked to (patho)physiological conditions, the pH synthetic aqueous chemistry of that metal ion was pursued in the presence of select α-hydroxycarboxylic acids. The organic acids were chosen so as to retain the α-hydroxycarboxylic acid moiety, while concurrently introducing hydrophobic methyl groups (HIBAH_2_, EHBAH_2_)) and a bulky cyclic aliphatic core containing alcoholic moieties at positions diametrically opposed to the α-hydroxycarboxylic acid moiety. The so-projected structural features provide a well-defined environment for the chelating anchor α-hydroxycarboxylic acid moiety to seek binding to Ti(IV) and afford soluble hybrid complex titanoforms. Based on the aforementioned systemic configuration, a pH-specific synthetic effort was developed in the framework of structural speciation, characterizing all such binary and ternary systems of metal ions with biologically physiological substrates investigated in our lab. The herein investigated binary Ti(IV)-(α-hydroxycarboxylic acid) systems, encompassing the α-hydroxycarboxylic acids α-hydroxy isobutyric acid (HIBAH_2_), D-quinic acid, and 2-ethyl-2-hydroxybutyric acid (ΕHΒAH_2_), led to the isolation of crystalline materials. The four Ti(IV)-hydroxycarboxylato complexes were fully characterized analytically and spectroscopically, with X-ray crystallography unequivocally establishing their solid-state structure. The new titanium(IV) materials (NH_4_)_2_[Ti(C_4_H_6_O_3_)_3_]·3.5H_2_O (**1**), (CH_6_N_3_)_2_[Ti(C_4_H_6_O_3_)_3_]∙1.5H_2_O (**2**), (NH_4_)_2_[Ti(C_7_H_10_O_6_)_3_]·2H_2_O (**3**), and [Ti(C_6_H_11_O_3_)_2_(C_6_H_10_O_3_)]·4.5H_2_O (**4**), as mentioned above, are all soluble in water. Considering the notion that solubility is strongly associated with bioavailability at the cellular level, the water-soluble nature of **1**–**4** arose as a very important property for their further potential biological interactions with components of living tissues and biological fluids.

With regard to their structural characteristics in the solid state, the complex assemblies in **1**–**4** exhibit interesting similarities and differences as well. In all structures delineated, (a) the associated complex assemblies are mononuclear, (b) Ti(IV) appears to be octahedrally coordinated by three ligands, (c) the bound ligands are singly and/or doubly deprotonated through their carboxylic and alcoholic moieties, (d) the deprotonated ligands act as metal ion chelators, thereby giving rise to the formation of five-membered metallacyclic rings, consequently providing stability to the Ti(IV) species, and (e) in the majority of the species studied, the use of base or base-acting agents has resulted in the emergence of cations capable of counteracting the negative charge arising on the assembled complex species isolated in the solid state.

In contrast to compounds **1**–**3**, the complex assembly in **4** is neutral. In the synthetic procedure of that complex, adjustment of pH to a specific value resulted in the isolation of a neutral species. Furthermore, the case of compound **4** is unique among the species studied in that the coordination sphere of Ti(IV) contains ligands of the same nature yet of a different deprotonation state, thereby reflecting the stability of the mononuclear assembly under the specific pH conditions. The ligand ΕHΒAH_2_ was found to be bound to the Ti(IV) center in the form of [C_6_H_11_O_3_]^−^ and [C_6_H_10_O_3_]^2−^, pointing out the two sites of deprotonation to the carboxylic acid and alcoholic moieties. Thus, the prospect of other species participating in the aqueous speciation scheme of the Ti(IV)-ΕHΒAH_2_ system is possible, with various degrees of deprotonation of the specific ligand(s) supporting the diverse nature of the metal ion complex assembly, bearing overall negative charge in line with the other species studied in this work and in the past.

With respect to the architectural arrangement of the structure in compounds **1**–**4**, the X-ray crystallographic study revealed the presence of a strong network of hydrogen bonds between the countercations, the lattice water molecules, and the organic binders (HIBA^2−^, D-quinato, and 2-ethyl-2-hydroxybutyrato ligands). As a consequence of the hydrogen-bonding network, 3D rigid lattices arose in all Ti(IV)-(α-hydroxycaboxylato) materials **1**–**4**.

Structural comparisons of the complex assemblies in **1**–**4** with previous species of aqueous binary Ti(IV)-ligand systems [32,33,34] reveal that all species exhibit a mononuclear Ti(IV)-O_6_ core. On the other hand, complexes **1**–**4** exhibit significant structural differences in comparison to previously reported dinuclear seven-coordinated Ti(IV)-citrato–peroxido complexes [26].

Given the ultimate goal of using the specific complexes in biological studies assessing their biochemical activity under pathophysiological conditions in humans (in the present case, diabetes mellitus, especially type 2, which is due to significant insulin resistance) [40], physicochemical characterization of the arisen species in the solid state (elemental analysis, FT-IR, TGA, and X-ray crystallography) was pursued into the solution state over time, with ESI-MS spectrometry defining the species arising upon dissolution of the requisite compounds to be employed in biological studies. Therefore, a complete profile of the compounds tested biologically was provided, thereby justifying further attempts to peruse their biological potential at the cellular and genetic level.

### 3.2. Biological Profile Studies

Critical prerequisites for the use of any metal-containing compound in studies investigating its potential induction of an effect in the cellular milieu are (a) solubility that begets bioavailability, which in turn promotes the chemistry of interactions with biological targets linked to the anticipated and/or observed phenotype, and (b) atoxicity, when it comes to meaningful effects supporting physiology. In the present study, all species **1**–**4** are soluble in water, and their physicochemical profile has been defined through solid-state–solution correlation studies (vide supra). To that end, two of the four compounds, i.e., **1** and **3**, were chosen for further biological testing due to their discrete yet representative structural chemical identity in the family of species synthesized.

Prior to the assessment of the potential biomimetic activity of both **1** and **3,** their cytotoxic profile was investigated, since low or zero toxicity is fundamentally significant and thus a desired prerequisite for further consideration. In so doing, both compounds were used for the treatment of 3T3-L1 pre-adipocytes and Saos-2 osteoblasts (Figure 6 and Figure 7). Both compounds appear to be non-toxic to the two types of cells, even at high concentrations (100 μΜ), over both short (24 h) and longer times (48 h) of incubation. The collective results, pertaining to cell survival, indicate that both compounds can be used at concentrations up to 100 μΜ, whereas no cell proliferation effect was observed. Moreover, both compounds seem to be less toxic to Saos-2 cells than in the case of pre-adipocytes, thus indicating a tissue-specific effect, especially at higher concentrations. Low cell toxicity is extremely significant, given that the ensuing differentiation protocols employed in both cases (adipogenesis and mineralization) project long-term periods of incubation and hence cell exposure to the Ti(IV)-complexes. In the current study, the employed complexes project no toxicity issues since they have been tested in two different cell types over long periods of exposure, with the results strongly suggesting that they do not affect cell function negatively for both osteoblasts and adipocytes.

Cell toxicity was also investigated in terms of cell migration since it is well established that even when a chemical factor does not appear to reduce cell survival, it could have a negative impact on physiological cell motility. For this, 3T3-L1 pre-adipocytes were employed, given that the specific cell line chemotactically exemplifies its migrating effect [27]. In the present study, an in vitro scratch assay was carried out in the presence of either **1** or **3**. As shown experimentally, both compounds do not seem to inhibit the physiological migration of the cells, although in the case of 50 μΜ concentrations, the migrating capacity of the cells is slightly inhibited compared to higher concentrations (100 μΜ).

Meanwhile, cell morphology results suggest that both compounds have no effect on cell physiology with regard to shape, appearance, color, adhesion, and confluency. The overall cytotoxic characterization of **1** and **3** formulates the biological activity profile of the title species toward 3T3-L1 and Saos-2 cells. In so doing, the arising (a) toxic profile of **1** and **3** provides the basis for well-defined and credible candidate biomimetic factors.

After having examined the cell toxicity of the complexes in terms of viability, migration, and morphology, both **1** and **3** were used to investigate their potential insulin-mimetic activity. In so doing, 3T3-L1 pre-adipocytes were differentiated into mature adipocytes in the presence of either **1** or **3**, following a standard differentiation protocol, by completely replacing insulin, in view of the fact that insulin serves as the key adipogenic agent. The results show that both complexes induce cell differentiation, when compared to untreated cells. In the case of **1**, at concentrations in the range 1–50 μM, the adipogenic activity seems to be concentration-dependent. However, it stands at no higher level than the effect seen with insulin (positive control of the assay). Only in combination with insulin does **1** induce the same adipogenic capacity, thereby indicating synergism and hence no competitive activity toward insulin. In the case of **3**, cell differentiation is again induced when insulin is totally replaced, but the effect is not concentration-dependent. Moreover, compound **3** does not seem to enhance insulin activity, in view of the fact that when **3** is used in combination with insulin at its highest concentration, the emerging adipogenic activity is equal to that of cells induced to differentiate only in the presence of **3**.

In an attempt to investigate the multifunctional biological/biomimetic profile of the title complexes, **1** and **3** were employed in experiments targeting the induction of cell mineralization. In so doing, Saos-2 cells were treated with various concentrations (1–100 μΜ) of **1** or **3.** A standard mineralization protocol was followed involving ascorbic acid and β-glycerophosphate as the positive control of the assay. Here, too, cells were successfully differentiated in all cases examined, with the results reflecting clear effects when compared to the positive control for both complexes. The highest effect observed in the case of **1** (10 μΜ) and **3** (100 μΜ) indicates (a) clear differentiation of the mineralization potential between the two compounds, and (b) enhancement of mineralization in both cases compared to the control, in the presence of the mineralization induction medium. Favorable osteogenic activity was further investigated in KS483 pre-osteoblasts. The effect of the various compounds on mineralization in KS483 cells is concentration-dependent compared to unstimulated cells. The maximum effect was observed in the case of compound **1** (up to ~47% at 100 μΜ).

It should be mentioned that the balance between adipogenesis and osteogenesis, especially when it comes to bone regeneration, arises as a significant factor in view of the fact that bone remodeling/regeneration is considered as a complex process. It has been shown both in vitro and in vivo that this process includes close interactions between different progenitor cell lineages, mainly osteo- and adipo-progenitor cells. At the clinical level, several studies support the fact that increased adipogenesis and fat accumulation in the bone marrow are correlated to decreased bone mineral density in the elderly and the osteoporotic population [41,42].

Taken together, the results of this study indicate that both **1** and **3** can serve as potentially insulin-mimetic agents (that can effectively replace insulin and/or enhance its action) with a favorable effect on bone tissue as well. The present study sets the basis for further investigation, both in vitro and in vivo, to uncover key markers, project interactions with specific molecular targets, and investigate mechanistic schemes of interacting molecules that support the present findings and identify the role of such compounds in the signaling pathways involved in normal and pathological processes (e.g., pertaining to safety and/or immunogenicity).

## 4. Methods and Materials

### 4.1. Material and Methods

All experiments were carried out in air. TiCl_4_, α-hydroxy isobutyric acid (HIBAH_2_), D-quinic acid (QA), 2-ethyl-2-hydroxy-butyric acid (EHBAH_2_), ammonia_,_ guanidinium carbonate, and ethanol were supplied by commercial sources (Alfa Aesar, Kandel, Germany; Merck SA, Darmstadt, Germany; VWR, Bridgeport, CT, USA). Ultrapure water was used in all experiments.

#### 4.1.1. Physical Measurements

A Thermo Electron Nicolet IR 200 FT-Infrared spectrometer, using KBr tablets, was used to obtain the FT-infrared spectra. Carbon, hydrogen, and nitrogen (%) determinations were recorded simultaneously on a Thermo Finnigan Flash EA 1112 CHNS elemental analyzer (Thermo Fisher Scientific, Waltham, MA, USA), fully automated and PC-controlled via the Eager 300 software. The analyzer operation involves dynamic flash combustion of the sample at 1800 °C, followed by reduction, trapping, complete GC separation, and product detection.

#### 4.1.2. Thermal Studies

A Perkin Elmer Pyris 1 Thermogravimetric Analysis (TGA) system was used for the thermogravimetric analysis of the compounds in an aerobic atmosphere. The sample mass was in the range 2–10 mg, and the heating rate was 5 °C/min, in the temperature range 25–900 °C. Sample weight loss was recorded continuously during the procedure, as a function of time or temperature, under dynamic conditions in the range of 25–900 °C.

#### 4.1.3. Mass Spectrometry Measurements (ESI-MS)

Electrospray ionization mass spectrometry (ESI-MS) infusion experiments were carried out on a ThermoFisher Scientific (Bremen, Germany) model LTQ Orbitrap Discovery MS. The free ligands α-hydroxy-isobutyric acid (HIBAH_2_) (L_1_H_2_ = (C_4_H_8_O_3_)), quinic acid (L_2_H_2_ = (C_7_H_12_O_6_)), and 2-ethyl-2-hydroxy-butyric acid (EHBAH_2_) (L_3_H_2_ = (C_6_H_12_O_3_)) and all titanium binary compounds (NH_4_)_2_[Ti(L_1_)_3_]·3.5H_2_O (**1**), (CH_6_N_3_)_2_[Ti(L_1_)_3_]∙1.5H_2_O (**2**), (NH_4_)_2_[Ti(L_2_)_3_]∙2H_2_O (**3**), and Ti(L_3_H)_2_(L_3_)·4.5H_2_O (**4**) were dissolved in water and introduced into the ESI source of the MS, through an integrated syringe pump, at a flow rate of 10 μL/min. The infusion experiments were run using a standard ESI source, operating in a positive ionization mode. Source operating conditions were a 3.7 kV spray voltage and a 300 °C heated capillary temperature.

### 4.2. Synthesis of Binary Ti(IV) Compounds

#### 4.2.1. (NH_4_)_2_[Ti(C_4_H_6_O_3_)_3_]·3.5H_2_O (**1**)

A quantity of α-hydroxy isobutyric acid (HIBAH_2_) (0.30 g, 3.0 mmol) was placed in a round-bottom flask and dissolved in 10 mL of H_2_O, under continuous stirring, at room temperature. The solution was placed in an ice–water bath for cooling. A solution of TiCl_4_ (0.10 mL, 1.0 mmol) was added to the resulting cold solution of the ligand, and stirring ensued for 15 min. The emerging reaction mixture was left to stir at room temperature, and then pH was adjusted by the gradual addition of an aqueous solution of ammonia (1:1) to a final value of 5. The resulting clear reaction solution was placed in a vial and left at room temperature to evaporate slowly. After 20 days, a colorless crystalline product appeared at the bottom of the vial; it was isolated by filtration and dried in the air. Yield 0.18 g (~39%). Anal. Calc. for **1**, (NH_4_)_2_[Ti(C_4_H_6_O_3_)_3_]^.^3.5H_2_O (C_12_H_33_N_2_O_12.5_Ti, M_r_ 453.30): C 31.76, H 7.28, N 6.18. Found: C 31.74, H 7.29, N 6.20.

#### 4.2.2. (CH_6_N_3_)_2_[Ti(C_4_H_6_O_3_)_3_]∙1.5H_2_O (**2**)

An amount of α-hydroxy isobutyric acid (HIBAH_2_) (0.30 g, 3.0 mmol) was dissolved in 10 mL of H_2_O, in a round-bottom flask, under stirring at room temperature. The solution was cooled in an ice–water bath. Subsequently, TiCl_4_ (0.10 mL, 1.0 mmol) was added under stirring. The solution was then stirred for 15 min at room temperature. As a next step, the addition of guanidinium carbonate adjusted the pH of the reaction mixture to a final value of 6.5. The arising reaction mixture was allowed to stir at room temperature. Upon standing for three weeks at room temperature, white needle-like crystals emerged at the bottom of the vial. The crystalline material was isolated by filtration and air-dried. Yield 0.18 g (~35%). Anal. Calc. for **2**, (CH_6_N_3_)_2_[Ti(C_4_H_6_O_3_)_3_]∙1.5H_2_O (C_14_H_33_N_6_O_10.5_Ti, M_r_ 501.35): C 33.67, H 6.61, N 16.84. Found: C 33.66, H 6.62, N 16.83.

#### 4.2.3. (NH_4_)_2_[Ti(C_7_H_10_O_6_)_3_]∙2H_2_O (**3**)

Method A: A quantity of D-quinic acid (0.40 g, 2.0 mmol) was placed in a round-bottom flask and dissolved under stirring, in 3 mL of H_2_O at room temperature. The solution was cooled into an ice–water bath. Next, a solution of TiCl_4_ (0.10 mL, 1.0 mmol) was added under stirring. The reaction mixture was then allowed to stir at room temperature for 30 min. Subsequently, aqueous ammonia (1:1) was added gradually to adjust the pH to a final value of 6.5. The emerging reaction solution was placed at 4 °C, and cold ethanol was added periodically. After two months, a colorless crystalline product was obtained at the bottom of the flask. The crystals were isolated by vacuum filtration and air-dried. Yield 0.18 g (22%). Anal. Calc. for **3**, (NH_4_)_2_[Ti(C_7_H_10_O_6_)_3_]∙2H_2_O (3) (C_21_H_42_N_2_O_20_Ti, M_r_ 690.35): C 36.50, H 6.00, N 4.06. Found: C 36.58, H 6.03, N 4.08.

Method Β: The reaction between TiCl_4_ and D-quinic acid, in a molar ratio 1:3, was run at a final pH value of 4 (adjustment with aqueous ammonia). The addition of ethanol at 4 °C for 2–3 months led to the isolation of the same crystalline material **3**. Yield 0.17g (~20%). The identity of the material was confirmed through FT-IR spectroscopy and X-ray unit cell parameter determination of single crystals emerging from the reaction mixtures.

#### 4.2.4. Ti(C_6_H_11_O_3_)_2_(C_6_H_10_O_3_)·4.5H_2_O (**4**)

A quantity of 2-ethyl-2-hydroxy-butyric acid (0.39 g, 3.0 mmol) (ligand) was placed in a round-bottom flask and dissolved in 15 mL of H_2_O. The ligand solution was left to stir at room temperature. Then, the clear solution was cooled in an ice–water bath, and a quantity of TiCl_4_ (0.10 mL, 1.0 mmol) was added under continuous stirring. The reaction mixture was left under stirring for 30 min, slowly returning to room temperature. Subsequently, the pH of the reaction mixture was adjusted by the gradual addition of an aqueous solution of ammonia (1:1) to a final value of 5. The final reaction solution was allowed to stand at room temperature for slow evaporation. After 25 days, a white crystalline product appeared at the bottom of the vial; it was isolated by filtration and dried in the air. Yield 0.14 g (~26%). Anal. Calc. for **4**, Ti(C_6_H_11_O_3_)_2_(C_6_H_10_O_3_)·4.5H_2_O (C_18_H_41_O_13.50_Ti, M_r_ 521.42): C 41.50, H 7.50. Found: C 41.48, H 7.47.

### 4.3. X-ray Crystal Structure Determination

Single crystals of compounds **1**–**3** were taken from the mother liquor and placed at room temperature on a Bruker Kappa APEX II diffractometer, equipped with a triumph monochromator using Mo Ka radiation (λ 0.71073 Å). In the case of compound **4**, a temperature of 130 K was achieved with a liquid nitrogen gas stream, using a Kryoflex 2 cryostat, and kept constant through data collection. Determination of unit cell dimensions took place using the angular settings of at least 135 high-intensity reflections (>10 σ(I)) in the range 20° < 2θ < 42°. Data collection was pursued using φ and ω scans. During data collection, no crystal decay was observed. The collected data were integrated with the Bruker SAINT software package (Version 8.34A) [43], using a narrow-frame algorithm. The numerical method SADABS, based on the crystal dimensions, was used for the absorption correction of the collected data [44]. Data refinement was performed using full matrix least-squares methods on *F*^2^, and all other calculations were performed using the Crystals version 14.61_build_6236 program package [45]. The structures were solved using the SUPERFLIP method [46] in the crystallographic package Crystals. All non-hydrogen atoms, except for the disordered solvent atoms, were anisotropically refined. All disordered non-hydrogen solvent atoms were properly treated. All non-disordered hydrogen atoms were located at their expected positions and refined using proper constraints. Disordered water hydrogen atoms were positioned to fulfill hydrogen-bonding demands. Finally, molecular illustrations were drawn with the Diamond 3.1 crystallographic package [47]. Crystallographic details for all compounds are summarized in Table 1 and Tables S1–S12.

### 4.4. Cell Cultures and Biological Tests

#### 4.4.1. Cultivation of 3T3-L1, Saos-2, and KS483 Cells

In the present study, three cell lines, namely (a) 3T3-L1 (mouse pre-adipocytes), (b) Saos-2 (osteosarcoma cell lines that display osteoblastic features), and (c) KS483 (mouse pre-osteoblastic cells) were employed. Cells were seeded in 75 cm^2^ cell culture flasks and incubated under appropriate conditions (5% CO_2_ at 37 °C and standard humidity) in Dulbecco’s modified Eagle’s medium (DMEM) (Sigma, Steinheim, Germany) or α minimum essential medium (αMEM) for KS483 and supplemented with 10% fetal bovine serum (FBS) (Biochrom, Berlin, Germany) and 1% penicillin–streptomycin (Biochrom, Berlin, Germany).

#### 4.4.2. Preparation of Ti(IV)-Compound Stock Solution

Fresh stock solutions of the title compounds were prepared in either DMEM (1% penicillin–streptomycin, 10% FBS) in the case of 3T3-L1 and Saos-2 treatments or αMEM (1% penicillin–streptomycin, 10% FBS) in the case of KS483 treatments, at a concentration of 1 mM, followed by sterile filtration. All tested compounds exhibited high water solubility. Final working concentrations were added directly to the cell cultures and incubated over the desired time periods according to protocols followed.

#### 4.4.3. Cell Viability–Growth Assay

To investigate the potential cytotoxic effects of the newly synthesized materials (ligands, binary titanium–ligand compounds), cells (3T3-L1 pre-adipocytes, Saos-2 osteoblasts) were seeded in 96-well plates (2500 cells/well) and treated with the title materials for 24 and 48 h. The assay is based on the quantitation of ATP present, which attests to the presence of metabolically active and hence viable cells. The reagent was added to the cell culture according to manufacturer instructions (volumetric reagent/supernatant ratio 1:1), without removing the supernatant, as described elsewhere [27]. The luminescence signal intensity, produced by the luciferase reaction, was determined using a Glomax 96 microplate luminometer (Promega Corporation, Madison, WI, USA). In the case of KS483 cells, cell viability was monitored simultaneously with the induction of the differentiation process.

#### 4.4.4. Cell Migration Assay

To investigate the potential cytotoxic profile of the newly synthesized materials, a cell migration assay was run. The potential inhibition of the endogenous motility of 3T3-L1 pre-adipocytes was evaluated using a 2D in vitro scratch assay. In this regard, cells were seeded in 35 mm cell culture dishes in DMEM and allowed to grow until 70–80% confluency had been achieved. Then, a scratch in the monolayer was made, over the entire diameter of each culture dish, using a sterile pipette tip (100 μL), and cells were incubated in the culture medium in the presence of final concentrations of 50 μΜ and 100 μΜ of the title compounds. Cells were visualized using an Axio Observer Z1 microscope, with a 10× phase contrast (Carl Zeiss, GmbH Lena, Germany). Images were captured, using an AxioCamHc camera, 24 h after the scratch had been made.

#### 4.4.5. Cell Biocompatibility—Morphology

Potential cytotoxic effects, in the presence of the materials tested, were also investigated with respect to cell morphology. To that end, both cell types were regularly examined with respect to shape, appearance, color, confluency, etc., to further confirm any aberration from the healthy status. Cells were visualized using an Axio Observer Z1 microscope, with a 10× phase contrast (Carl Zeiss, GmbH Lena, Germany). Images were captured, using an AxioCamHc camera, at several time points (prior to and after treatment).

#### 4.4.6. Induction of Adipogenesis with Ti(IV)-Complexes In Vitro

3T3-L1 pre-adipocytes were differentiated into mature adipocytes, following the standard differentiation protocol (vide supra) as described elsewhere [48]. 3T3-L1 fibroblast-like cells were treated with either 10 ng/mL of insulin and/or selected well-defined titanium compounds (1–50 μΜ). In all experiments run, the insulin group was used as a positive control. A control group with no treated cells (without insulin or titanium complexes) was also included. On the 8th day of the differentiation process, cell differentiation was assessed with oil red O staining. All tests were carried out at least in triplicate.

#### 4.4.7. Oil Red O Staining

Successful cell differentiation into mature adipocytes was evaluated through oil red O staining, which was performed on the 8th day of the differentiation process. For that purpose, cells were washed with PBS (1X, pH 7.4) and fixed with 4% formalin for 20 min. Then, cells were washed with sterile doubly deionized water (ddH_2_O) and treated with oil red O working solution for 15 min at room temperature. Subsequently, cells were washed with sterile ultrapure water and stained with hematoxylin for 1 min at room temperature. Cells were visualized using an Axio Observer Z1 microscope, with a 10× and 40× phase contrast objective (Carl Zeiss, GmbH Jena, Germany). Images were captured on an AxioCam Hc camera. All tests were carried out at least in triplicate. Isopropanol was used to elute oil red O staining from the lipid droplets, and then spectrophotometric semi-quantification was performed at 518 nm.

#### 4.4.8. In Vitro Mineralization

In an effort to assess the effect of the newly synthesized materials on bone formation, an in vitro mineralization assay was carried out. To this end, KS483 pre-osteoblastic cells were used to differentiate them into mature osteoblasts. In brief, cells were seeded in twelve-well plates (at a density of 5 × 10^4^ cells per well). Three days after plating, cells reached confluency and were subsequently induced to differentiate by adding 50 mM ascorbic acid (Applichem, Darmstadt, Germany) to the culture, in the presence or absence of various concentrations (1, 10, 50, 100 μM) of the tested compounds. The medium along with substances was refreshed every 3rd day for 27 consecutive days in total. In the case of Saos-2, the same aforementioned procedure was followed, with the medium composed of 5 mM β-glycerophosphate, 50 mM ascorbic acid, and 10 nM dexamethasone. The medium along with substances was refreshed every 3rd day for 14 consecutive days in total.

#### 4.4.9. Alizarin Red Staining

To confirm and semi-quantitatively assess success in mineralization, the cultured cells were rinsed with PBS, followed by fixation with 10% formalin for 15 min and subsequent staining with Alizarin Red-S (solution 2%, pH 4.8) (Sigma) for 15 min. After staining, cells were first rinsed with PBS and then incubated with 10% acetic acid for 30 min. Cells were subsequently scraped from the plate, heated at 85 °C for 10 min, and then kept on ice for an additional 10 min. A volume of 200 μL of each extraction was incubated with 75 μL of 10% ammonia, and the colored product was measured at 550 nm on an Elisa reader. Mineralization was expressed as absorbance per well (OD 550 of treated cells/OD 550 of untreated cells × 100).

#### 4.4.10. Statistical Analysis

The obtained data were presented as average and standard error mean (SEM) values of triplicate sets of independent measurements. Mean survival rates and SEMs were calculated for each individual group. Absolute survival rates were calculated for each control group, and one-way analysis of variance (ANOVA) was performed for all pair comparisons, followed by post hoc analyses (Tukey) using GraphPad Prism v.6. Degrees of significance were assessed using different rating values: * *p* < 0.05 (significant), ** *p* < 0.01 (highly significant), *** *p* < 0.001 (extremely significant), **** *p* ≤ 0.0001 (extremely significant), and non-significant (*p* > 0.05).

## 5. Conclusions

Prompted by the challenge to develop soluble and bioavailable forms of Ti(IV) that could be used to probe that metal ion’s adipogenic potential in diabetes mellitus type 2, the pH-specific aqueous synthetic chemistry of binary Ti(IV)-(α-hydroxycarboxylic acid) systems was pursued. The emerging new crystalline materials **1**–**4** were physicochemically characterized in the solid state and in solution, thereby providing a comprehensive bioprofile, further justifying the employment of select species in the intended biological studies. The ensuing biological studies in three cell lines led to the formulation of a well-defined toxicity profile for the title compounds. Further cell differentiation studies, in the presence of the atoxic binary Ti(IV) compounds **1** and **3,** provided definitive clues on the involvement of that metal ion in processes leading to mature adipocytes capable of glucose catabolism. The solid-state–solution correlation studies of the binary Ti(IV) compounds reflect a structure-specific biological participation of Ti(IV) in subcellular activity linked to pre-adipocyte differentiation and adipocyte maturation, thus presenting the salient features of that early transition metal ion that render it efficient in the investigated bioprocess. The collective interdisciplinary work justifies for the first time the phenotypic involvement of multifunctional Ti(IV) complex forms in cellular differentiation processes, expressly projecting (a) insulin mimetic behavior toward adipogenesis, while concurrently exhibiting a favorable effect during osteogenesis, and (b) events supporting further perusal (both in vitro and in vivo) into specific molecular interactions exemplifying the phenotypic behavior observed and hence the potential use of such species as future metallopharmaceuticals in diabetes mellitus type 2.

## Figures and Tables

**Table 1:** Crystal data and experimental details for (NH_4_)_2_[Ti(C_4_H_6_O_3_)_3_]·3.5H_2_O (**1**), (CH_6_N_3_)_2_[Ti(C_4_H_6_O_3_)_3_]∙1.5H_2_O (**2**), (NH_4_)_2_[Ti(C_7_H_10_O_6_)_3_]·2H_2_O (**3**), and [Ti(C_6_H_11_O_3_)_2_(C_6_H_10_O_3_)]·4.5H_2_O (**4**).

Compound	1	2	3	4
Chemical formula	C_24_H_66_N_4_O_25_Ti_2_	C_28_H_66_N_12_O_21_Ti_2_	C_21_H_42_N_2_O_20_Ti	C_18_H_41_O_13.50_Ti
*M*_r_	906.60	1002.70	690.35	521.42
Crystal system	Triclinic	Monoclinic	Cubic	Monoclinic
Space group	*P*ī	*P*2_1_/*n*	*P*2_1_3	*C*2/*c*
Temperature (K)	295	295	295	130
*a* (Å)	10.530 (6)	21.458 (7)	15.1689 (6)	17.017 (2)
*b* (Å)	11.531 (6)	10.212 (3)	15.1689 (6)	11.3810 (15)
*c* (Å)	18.946 (11)	23.248 (7)	15.1689 (6)	27.743 (3)
α (°)	89.09 (3)	90	90	90
β (°)	89.82 (3)	94.828 (8)	90	101.800 (3)
γ (°)	80.80 (3)	90	90	90
*V* (Å^3^)	2270 (2)	5076 (3)	3490.3 (4)	5259.3 (11)
*Z*	2	4	4	8
Radiation type	Mo *K*α	Mo *K*α	Mo *K*α	Mo *K*α
µ (mm^−1^)	0.43	0.39	0.32	0.39
Crystal size (mm)	0.26 × 0.16 × 0.13	0.22 × 0.13 × 0.11	0.19 × 0.16 × 0.11	0.11 × 0.09 × 0.06
*T*_min_, *T*_max_	0.93, 0.95	0.95, 0.96	0.95, 0.97	0.97, 0.98
**No. of reflections**				
measured	69,918	63,319	13,087	28,368
independent	9847	9628	2392	5231
observed [*I* > 2.0σ(*I*)]	8419	6040	1927	4113
No. of parameters	499	568	151	334
No. of restraints	0	0	7	18
*R*_int_	0.015	0.057	0.056	0.018
(sinθ/λ)_max_ (Å^−1^)	0.643	0.613	0.625	0.623
**Refinement**				
*R*[*F*^2^ > 2σ(*F*^2^)]	0.041	0.052	0.053	0.042
*R_w_*(*F*^2^)	0.062	0.088	0.137	0.082
*S*	1.00	1.00	1.00	1.00
Δρ_max_, Δρ_min_ (e Å^−3^)	0.26, −0.34	0.53, −0.39	0.49, −0.29	0.65, −0.53
Absolute structure parameter			−0.02 (6)	

**Table 2:** Bond lengths (Å) and angles (deg) for (NH_4_)_2_[Ti(C_4_H_6_O_3_)_3_]·3.5H_2_O (**1**), (CH_6_N_3_)_2_[Ti(C_4_H_6_O_3_)_3_]∙1.5H_2_O (**2**), (NH_4_)_2_[Ti(C_7_H_10_O_6_)_3_]·2H_2_O (**3**), and [Ti(C_6_H_11_O_3_)_2_(C_6_H_10_O_3_)]·4.5H_2_O (**4**).

**Bond Length (Å)**							
**1**		**2**		3 *		**4**	
Ti(1)—O(1)	2.0378 (16)	Ti(1)—O(1)	2.065 (2)	Ti(1)—O(1)	2.039 (3)	Ti(1)—O(1)	2.0605 (16)
Ti(1)—O(3)	1.8742 (15)	Ti(1)—O(3)	1.856 (2)	Ti(1)—O(3)	1.866 (2)	Ti(1)—O(3)	1.8675 (16)
Ti(1)—O(4)	2.0644 (16)	Ti(1)—O(4)	2.070 (2)			Ti(1)—O(4)	2.0340 (16)
Ti(1)—O(6)	1.8639 (15)	Ti(1)—O(6)	1.861 (2)			Ti(1)—O(6)	1.8582 (16)
Ti(1)—O(7)	2.0363 (17)	Ti(1)—O(7)	2.052 (2)			Ti(1)—O(7)	2.0445 (16)
Ti(1)—O(9)	1.8703 (15)	Ti(1)—O(9)	1.860 (2)			Ti(1)—O(9)	1.8493 (16)
Ti(2)—O(10)	2.0546 (17)	Ti(2)—O(10)	2.048 (2)				
Ti(2)—O(12)	1.8679 (15)	Ti(2)—O(12)	1.878 (2)				
Ti(2)—O(13)	2.0414 (17)	Ti(2)—O(13)	2.061 (2)				
Ti(2)—O(15)	1.8666 (15)	Ti(2)—O(15)	1.874 (2)				
Ti(2)—O(16)	2.0474 (17)	Ti(2)—O(16)	2.072 (2)				
Ti(2)—O(18)	1.8817 (15)	Ti(2)—O(18)	1.827 (2)				
**Angles (°)**							
O(1)—Ti(1)—O(3)	78.95 (6)	O(1)—Ti(1)—O(3)	79.17 (9)	O(1) ^i^—Ti(1)—O(1) ^ii^	83.76 (13)	O(1)—Ti(1)—O(3)	77.99 (6)
O(1)—Ti(1)—O(4)	80.72 (7)	O(1)—Ti(1)—O(4)	80.80 (8)	O(1) ^i^—Ti(1)—O(3) ^i^	78.57 (11)	O(1)—Ti(1)—O(4)	84.76 (7)
O(3)—Ti(1)—O(4)	158.42 (6)	O(3)—Ti(1)—O(4)	159.23 (9)	O(1) ^ii^—Ti(1)—O(3) ^i^	159.59 (11)	O(3)—Ti(1)—O(4)	103.72 (7)
O(1)—Ti(1)—O(6)	101.93 (7)	O(1)—Ti(1)—O(6)	100.37 (10)	O(1) ^i^—Ti(1)—O(3) ^ii^	104.19 (11)	O(1)—Ti(1)—O(6)	160.50 (7)
O(3)—Ti(1)—O(6)	98.05 (7)	O(3)—Ti(1)—O(6)	99.60 (10)	O(3) ^i^—Ti(1)—O(3) ^ii^	95.87 (11)	O(3)—Ti(1)—O(6)	95.93 (7)
O(4)—Ti(1)—O6)	79.15 (7)	O(4)—Ti(1)—O(6)	78.67 (9)			O(4)—Ti(1)—O(6)	78.66 (7)
O(1)—Ti(1)—O(7)	83.54 (7)	O(1)—Ti(1)—O(7)	81.86 (9)			O(1)—Ti(1)—O(7)	85.08 (7)
O(3)—Ti(1)—O(7)	103.48 (8)	O(3)—Ti(1)—O(7)	98.51 (9)			O(3)—Ti(1)—O(7)	161.19 (7)
O(4)—Ti(1)—O(7)	81.22 (8)	O(4)—Ti(1)—O(7)	83.97 (8)			O(4)—Ti(1)—O(7)	82.60 (7)
O(6)—Ti(1)—O(7)	158.44 (6)	O(6)—Ti(1)—O(7)	161.85 (9)			O(6)—Ti(1)—O(7)	102.71 (7)
O(1)—Ti(1)—O(9)	160.26 (5)	O(1)—Ti(1)—O(9)	160.92 (9)			O(1)—Ti(1)—O(9)	103.53 (7)
O(3)—Ti(1)—O(9)	95.52 (8)	O(3)—Ti(1)—O(9)	101.20 (9)			O(3)—Ti(1)—O(9)	97.60 (7)
O(4)—Ti(1)—O(9)	106.06 (6)	O(4)—Ti(1)—O(9)	99.53 (9)			O(4)—Ti(1)—O(9)	158.35 (7)
O(6)—Ti(1)—O(9)	97.59 (7)	O(6)—Ti(1)—O(9)	98.37 (10)			O(6)—Ti(1)—O(9)	95.60 (7)
O(7)—Ti(1)—O(9)	79.33 (6)	O(7)—Ti(1)—O(9)	79.22 (9)			O(7)—Ti(1)—O(9)	78.31 (7)
O(10)—Ti(2)—O(12)	78.46 (6)	O(10)—Ti(2)—O(12)	78.78 (9)				
O(10)—Ti(2)—O(13)	81.99 (8)	O(10)—Ti(2)—O(13)	80.55 (9)				
O(12)—Ti(2)—O(13)	158.67 (6)	O(12)—Ti(2)—O(13)	100.63 (10)				
O(10)—Ti(2)—O(15)	104.53 (7)	O(10)—Ti(2)—O(15)	158.26 (10)				
O(12)—Ti(2)—O(15)	96.88 (7)	O(12)—Ti(2)—O(15)	99.05 (10)				
O(13)—Ti(2)—O(15)	79.81 (7)	O(13)—Ti(2)—O(15)	78.60 (9)				
O(10)—Ti(2)—O(16)	83.22 (7)	O(10)—Ti(2)—O(16)	82.84 (9)				
O(12)—Ti(2)—O16)	101.00 (7)	O(12)—Ti(2)—O(16)	160.96 (9)				
O(13)—Ti(2)—O(16)	84.96 (8)	O(13)—Ti(2)—O(16)	81.08 (9)				
O(15)—Ti(2)—O(16)	161.65 (6)	O(15)—Ti(2)—O(16)	99.87 (9)				
O(10)—Ti(2)—O(18)	160.75 (6)	O(10)—Ti(2)—O(18)	99.39 (10)				
O(12)—Ti(2)—O(18)	98.69 (8)	O(12)—Ti(2)—O(18)	99.36 (10)				
O(13)—Ti(2)—O(18)	102.58 (7)	O(13)—Ti(2)—O(18)	159.58 (9)				
O(15)—Ti(2)—O(18)	94.70 (7)	O(15)—Ti(2)—O(18)	102.29 (10)				
O(16)—Ti(2)—O(18)	78.62 (6)	O(16)—Ti(2)—O(18)	78.67 (9)				

* (^i^) −*z* + 3/2, −*x* + 1, *y* + 1/2; (^ii^) −*y* + 1, *z* − 1/2, −*x* + 3/2.

## Data Availability

The data and relevant material used and/or analyzed during the current study are available from the corresponding author on reasonable request.

## Supplementary Materials

The following supporting information can be downloaded at: https://www.mdpi.com/article/10.3390/ijms241411865/s1. CCDC 1940113 (**1**), 1940114 (**2**), 1940115 (**3**), and 1940116 (**4**) contain the supplementary crystallographic data for this paper. These data can be obtained free of charge via www.ccdc.cam.ac.uk/data_request/cif (accessed on 10 June 2023), or by emailing data_request@ccdc.cam.ac.uk, or by contacting The Cambridge Crystallographic Data Centre, 12 Union Road, Cambridge CB2 1EZ, UK; fax: +44 1223 336033. References [49,50,51,52,53] are cited in the supplementary materials.

## Abbreviations

ANOVA	One-way analysis of variance
DMEM	Dulbecco’s modified Eagle’s medium
EHBAH_2_	2-Ethyl-2-hydroxy-butyric acid
ESI-MS	Electron spray ionization mass spectrometry
FBS	Fetal bovine serum
FT-IR	Fourier transform infrared
HIBAH_2_	α-Hydroxy isobutyric acid
αMEM	α minimum essential medium
QA	Quinic acid
SEM	Standard error mean
TGA	Thermogravimetric analysis
