# Supplementary material for: Structural Speciation of Ti(IV)-(α-Hydroxycarboxylic Acid) Complexes in Metabolism-Related (Patho)Physiology—In Vitro Approaches to (Pre)Adipocyte Differentiation and Mineralization
Source: Int J Mol Sci. 2023 Jul 24;24(14):11865. doi: 10.3390/ijms241411865 (PMC10380816; doi:10.3390/ijms241411865)
Supplement: Supplementary file 1 [file ijms-24-11865-s001.zip › Final cifs and checkcifs/ki1138teliko comp4.pdf]

Table S10
Experimental details

Crystal data	
Chemical formula	$C_{18}H_{41}O_{13.50}Ti$
M_r	521.42
Crystal system, space group	Monoclinic, *C*2/*c*
Temperature (K)	130
a, *b*, *c* (Å)	17.017 (2), 11.3810 (15), 27.743 (3)
β (°)	101.800 (3)
V (Å^3)	5259.3 (11)
Z	8
Radiation type	Mo *K*α
μ (mm^{-1})	0.39
Crystal size (mm)	0.11 × 0.09 × 0.06
Data collection	
Diffractometer	Bruker Kappa Apex2
Absorption correction	Numerical<br>Analytical Absorption (De Meulenaer & Tompa, 1965)
T_{min}, T_{max}	0.97, 0.98
No. of measured, independent and observed [$I > 2.0\sigma(I)$] reflections	28368, 5231, 4113
R_{int}	0.017
$(\sin \theta/\lambda)_{max}$ (Å^{-1})	0.623
Refinement	
$R[F^2 > 2\sigma(F^2)]$, $wR(F^2)$, S	0.042, 0.082, 1.00
No. of reflections	4113
No. of parameters	334
No. of restraints	18
H-atom treatment	H atoms treated by a mixture of independent and constrained refinement
$\Delta\rho_{max}$, $\Delta\rho_{min}$ (e Å^{-3})	0.65, −0.53

Computer programs: Apex2 (Bruker AXS, 2006), *SUPERFLIP* (Palatinus & Chapuis, 2007), *CRYSTALS* (Betteridge *et al.*, 2003), *CAMERON* (Watkin *et al.*, 1996).

Table S11
Selected geometric parameters (Å, º)

Ti1—O1	2.0605 (16)	C1—C2	1.554 (3)
Ti1—O3	1.8675 (16)	C2—C3	1.499 (3)
Ti1—O4	2.0340 (16)	C3—C4	1.509 (4)
Ti1—O6	1.8582 (16)	C5—C6	1.529 (4)
Ti1—O7	2.0445 (16)	C7—C8	1.510 (4)
Ti1—O9	1.8493 (16)	C8—C11	1.466 (4)
O1—C1	1.272 (3)	C8—C19	1.4991 (10)
O2—C1	1.227 (3)	C9—C10	1.5005 (10)
O3—C2	1.410 (3)	C11—C21	1.4997 (10)
O4—C7	1.272 (3)	C13—C14	1.525 (3)
O5—C7	1.227 (3)	C14—C15	1.517 (4)
O6—C8	1.389 (3)	C14—C17	1.507 (4)
O7—C13	1.303 (3)	C15—C16	1.504 (4)
O8—C13	1.233 (3)	C17—C22	1.4987 (10)
O9—C14	1.417 (3)	C19—C20	1.4999 (10)
O1—Ti1—O3	77.99 (6)	O3—C2—C3	110.1 (2)
O1—Ti1—O4	84.76 (7)	C2—C3—C4	115.9 (2)
O3—Ti1—O4	103.72 (7)	C2—C5—C6	115.4 (2)
O1—Ti1—O6	160.50 (7)	O4—C7—O5	125.8 (2)
O3—Ti1—O6	95.93 (7)	O4—C7—C8	113.0 (2)
O4—Ti1—O6	78.66 (7)	O5—C7—C8	121.1 (2)
O1—Ti1—O7	85.08 (7)	C7—C8—O6	109.3 (2)
O3—Ti1—O7	161.19 (7)	C7—C8—C11	117.0 (3)
O4—Ti1—O7	82.60 (7)	O6—C8—C11	110.7 (2)
O6—Ti1—O7	102.71 (7)	C7—C8—C19	114.6 (3)
O1—Ti1—O9	103.53 (7)	O6—C8—C19	124.2 (3)
O3—Ti1—O9	97.60 (7)	C11—C8—C19	77.9 (3)
O4—Ti1—O9	158.35 (7)	C8—C9—C10	127.8 (5)
O6—Ti1—O9	95.60 (7)	C8—C11—C21	140.3 (4)
O7—Ti1—O9	78.31 (7)	O7—C13—O8	122.7 (2)
Ti1—O1—C1	117.07 (14)	O7—C13—C14	114.37 (19)
Ti1—O3—C2	124.39 (13)	O8—C13—C14	122.9 (2)
Ti1—O4—C7	117.73 (15)	C13—C14—O9	106.17 (18)
Ti1—O6—C8	121.24 (15)	C13—C14—C15	110.5 (2)
Ti1—O7—C13	116.83 (14)	O9—C14—C15	109.9 (2)
Ti1—O9—C14	123.86 (14)	C13—C14—C17	108.8 (2)
O1—C1—O2	124.4 (2)	O9—C14—C17	111.0 (2)
O1—C1—C2	115.35 (19)	C15—C14—C17	110.4 (2)

O2—C1—C2	120.3 (2)	C14—C15—C16	114.5 (2)
C1—C2—O3	105.14 (18)	C14—C17—C22	125.1 (3)
C1—C2—C3	110.2 (2)	C8—C19—C20	99.9 (4)

Table S12
Hydrogen-bond geometry (Å, º)

D—H···*A*	*D*—H	H···*A*	*D*···*A*	*D*—H···*A*
O3—H31···O14	0.83	2.14	2.894 (5)	150
C20—H91···C11	1.08	2.54	3.252 (5)	123
C20—H92···O6	0.76	2.45	2.996 (5)	129
O9—H94···O14	0.82	2.41	3.003 (5)	130
C10—H102···C18	0.96	1.96	2.882 (5)	160
O10—H106···O11	0.82	2.05	2.848 (5)	166
O10—H107···O12^{i}	0.82	1.97	2.768 (5)	165
O11—H111···O2^{ii}	0.82	1.87	2.684 (5)	168
O11—H112···O4	0.82	2.00	2.819 (5)	173
O12—H126···O2^{ii}	0.82	2.09	2.844 (5)	154
O12—H127···O7	0.82	2.04	2.830 (5)	160
O13—H131···O11	0.83	2.18	2.897 (5)	144
O13—H132···O8^{iii}	0.83	2.01	2.791 (5)	156
C18—H183···O6	0.96	2.59	3.342 (5)	135
O14—H143···O6	0.82	2.14	2.884 (5)	151
C18—H172···C13	0.77	2.47	3.186 (5)	155
C18—H172···C14	0.77	1.99	2.656 (5)	145
C18—H172···C17	0.77	0.95	1.499 (5)	121
C22—H174···C14	0.78	1.99	2.667 (5)	146
C22—H174···C15	0.78	2.55	3.296 (5)	161
C22—H174···C17	0.78	0.95	1.499 (5)	120

Symmetry codes: (i) $-x+3/2, y-1/2, -z+3/2$; (ii) $-x+3/2, y+1/2, -z+3/2$; (iii) $-x+2, y, -z+3/2$.

Supporting information

Crystallographic data

Acknowledgements
Funding information
References

43. Bruker Analytical X-ray Systems, Inc. Apex2, Version 2 User Manual, M86-E01078; Bruker Analytical X-ray Systems, Inc: Madison, WI, USA, 2006.

46. Betteridge, P.W.; Carruthers, J.R.; Cooper, R.I.; Prout, K.; Watkin, D.J.J. CRYSTALS version 12: software for guided crystal structure analysis. Appl. Cryst. 2003, 36, 1487. https://doi.org/10.1107/S0021889803021800.

49. De Meulenaer, J.; Tompa, H. The absorption correction in crystal structure analysis. *Acta Cryst.* **1965**, *19*, 1014-1018. https://doi.org/10.1107/S0365110X65004802.
50. Prince, E. *Mathematical Techniques in Crystallography and Materials Science*; Springer: New York, NY, USA, 1982.
51. Watkin, D.J. The control of difficult refinements. *Acta Cryst.* **1994**, A*50*, 411–437. https://doi.org/10.1107/S0108767393012784.
52. Watkin, D.J.; Prout, C.K.; Pearce, L.J. *CAMERON*; Chemical Crystallography Laboratory: Oxford, UK, 1996.
53. Flack, H.D. On enantiomorph-polarity estimation. *Acta Cryst.* **1983**, A*39*, 876–881. https://doi.org/10.1107/S0108767383001762.
