# Supplementary material for: Structural Speciation of Ti(IV)-(α-Hydroxycarboxylic Acid) Complexes in Metabolism-Related (Patho)Physiology—In Vitro Approaches to (Pre)Adipocyte Differentiation and Mineralization
Source: Int J Mol Sci. 2023 Jul 24;24(14):11865. doi: 10.3390/ijms241411865 (PMC10380816; doi:10.3390/ijms241411865)

checkCIF/PLATON report

Structure factors have been supplied for datablock(s) I

THIS REPORT IS FOR GUIDANCE ONLY. IF USED AS PART OF A REVIEW PROCEDURE FOR PUBLICATION, IT SHOULD NOT REPLACE THE EXPERTISE OF AN EXPERIENCED CRYSTALLOGRAPHIC REFEREE.

No syntax errors found. CIF dictionary Interpreting this report

Datablock: I

```
Bond precision:    C-C = 0.0036 A          Wavelength=0.71073

Cell:              a=17.017(2)     b=11.3810(15)        c=27.743(3)
                   alpha=90        beta=101.800(3)      gamma=90
Temperature:       130 K

                 Calculated                   Reported
Volume           5259.5(11)                   5259.3(11)
Space group      C 2/c                        C 1 2/c 1
Hall group       -C 2yc                       ?
Moiety formula   2(C18 H32 O9 Ti), 9(H2 O)    C18 H41 O13.50 Ti1
Sum formula      C36 H82 O27 Ti2              C18 H41 O13.50 Ti1
Mr               1042.76                      521.42
Dx,g cm-3        1.317                        1.317
Z                4                            8
Mu (mm-1)        0.385                        0.385
F000             2232.0                       2232.0
F000'            2235.63
h,k,lmax         21,14,34                     21,14,34
Nref             5328                         5231
Tmin,Tmax        0.959,0.977                  0.970,0.980
Tmin'            0.959

Correction method= # Reported T Limits: Tmin=0.970 Tmax=0.980
AbsCorr = NUMERICAL

Data completeness= 0.982            Theta(max)= 26.270

R(reflections)= 0.0424( 4113)                          wR2(reflections)=
                                                       0.0832( 4113)

S = 1.000                  Npar= 334
```

The following ALERTS were generated. Each ALERT has the format
test-name_ALERT_alert-type_alert-level.
Click on the hyperlinks for more details of the test.

Alert level C

```
PLAT041_ALERT_1_C Calc. and Reported SumFormula    Strings  Differ     Please Check
PLAT042_ALERT_1_C Calc. and Reported MoietyFormula Strings  Differ     Please Check
PLAT737_ALERT_1_C D...A   Calc  2.8944(15), Rep    2.894(5) ......        3.3 s.u.-R
              O3       -O14             1_555   1_555 ........     #    1 Check
PLAT737_ALERT_1_C D...A   Calc  3.0026(17), Rep    3.003(5) ......        2.9 s.u.-R
              O9       -O14             1_555   1_555 ........     #    4 Check
PLAT737_ALERT_1_C D...A   Calc  2.8837(17), Rep    2.884(5) ......        2.9 s.u.-R
              O14      -O6              1_555   1_555 ........     #   15 Check
PLAT911_ALERT_3_C Missing FCF Refl Between Thmin & STh/L=    0.600         20 Report
```

Alert level G

```
PLAT002_ALERT_2_G Number of Distance or Angle Restraints on AtSite         11 Note
PLAT003_ALERT_2_G Number of Uiso or Uij Restrained non-H Atoms ...          3 Report
PLAT007_ALERT_5_G Number of Unrefined Donor-H Atoms .............          12 Report
PLAT045_ALERT_1_G Calculated and Reported Z Differ by a Factor ...      0.500 Check
PLAT093_ALERT_1_G No s.u.'s on H-positions, Refinement Reported as      mixed Check
PLAT300_ALERT_4_G Atom Site Occupancy of C9         Constrained at        0.5 Check
PLAT300_ALERT_4_G Atom Site Occupancy of C10        Constrained at        0.5 Check
PLAT300_ALERT_4_G Atom Site Occupancy of C12        Constrained at        0.5 Check
PLAT300_ALERT_4_G Atom Site Occupancy of C18        Constrained at        0.5 Check
PLAT300_ALERT_4_G Atom Site Occupancy of C19        Constrained at        0.5 Check
PLAT300_ALERT_4_G Atom Site Occupancy of C20        Constrained at        0.5 Check
PLAT300_ALERT_4_G Atom Site Occupancy of C21        Constrained at        0.5 Check
PLAT300_ALERT_4_G Atom Site Occupancy of C22        Constrained at        0.5 Check
PLAT300_ALERT_4_G Atom Site Occupancy of H91        Constrained at        0.5 Check
PLAT300_ALERT_4_G Atom Site Occupancy of H92        Constrained at        0.5 Check
PLAT300_ALERT_4_G Atom Site Occupancy of H101       Constrained at        0.5 Check
PLAT300_ALERT_4_G Atom Site Occupancy of H102       Constrained at        0.5 Check
PLAT300_ALERT_4_G Atom Site Occupancy of H103       Constrained at        0.5 Check
PLAT300_ALERT_4_G Atom Site Occupancy of H113       Constrained at        0.5 Check
PLAT300_ALERT_4_G Atom Site Occupancy of H114       Constrained at        0.5 Check
PLAT300_ALERT_4_G Atom Site Occupancy of H115       Constrained at        0.5 Check
PLAT300_ALERT_4_G Atom Site Occupancy of H116       Constrained at        0.5 Check
PLAT300_ALERT_4_G Atom Site Occupancy of H121       Constrained at        0.5 Check
PLAT300_ALERT_4_G Atom Site Occupancy of H122       Constrained at        0.5 Check
PLAT300_ALERT_4_G Atom Site Occupancy of H123       Constrained at        0.5 Check
PLAT300_ALERT_4_G Atom Site Occupancy of H171       Constrained at        0.5 Check
PLAT300_ALERT_4_G Atom Site Occupancy of H172       Constrained at        0.5 Check
PLAT300_ALERT_4_G Atom Site Occupancy of H173       Constrained at        0.5 Check
PLAT300_ALERT_4_G Atom Site Occupancy of H174       Constrained at        0.5 Check
PLAT300_ALERT_4_G Atom Site Occupancy of H182       Constrained at        0.5 Check
PLAT300_ALERT_4_G Atom Site Occupancy of H183       Constrained at        0.5 Check
PLAT300_ALERT_4_G Atom Site Occupancy of H184       Constrained at        0.5 Check
PLAT300_ALERT_4_G Atom Site Occupancy of H191       Constrained at        0.5 Check
PLAT300_ALERT_4_G Atom Site Occupancy of H192       Constrained at        0.5 Check
PLAT300_ALERT_4_G Atom Site Occupancy of H201       Constrained at        0.5 Check
PLAT300_ALERT_4_G Atom Site Occupancy of H202       Constrained at        0.5 Check
PLAT300_ALERT_4_G Atom Site Occupancy of H203       Constrained at        0.5 Check
PLAT300_ALERT_4_G Atom Site Occupancy of H211       Constrained at        0.5 Check
```

```
PLAT300_ALERT_4_G Atom Site Occupancy of H212       Constrained at        0.5 Check
PLAT300_ALERT_4_G Atom Site Occupancy of H213       Constrained at        0.5 Check
PLAT300_ALERT_4_G Atom Site Occupancy of H221       Constrained at        0.5 Check
PLAT300_ALERT_4_G Atom Site Occupancy of H223       Constrained at        0.5 Check
PLAT300_ALERT_4_G Atom Site Occupancy of H224       Constrained at        0.5 Check
PLAT300_ALERT_4_G Atom Site Occupancy of H143       Constrained at        0.5 Check
PLAT300_ALERT_4_G Atom Site Occupancy of H144       Constrained at        0.5 Check
PLAT301_ALERT_3_G Main Residue  Disorder .............(Resd  1  )        14% Note
PLAT769_ALERT_4_G CIF Embedded explicitly supplied scattering data     Please Note
PLAT808_ALERT_5_G No Parseable SHELXL Style Weighting Scheme Found     Please Check
PLAT860_ALERT_3_G Number of Least-Squares Restraints ............          18 Note
PLAT882_ALERT_1_G No Datum for _diffrn_reflns_av_unetI/netI ......     Please Do !
PLAT910_ALERT_3_G Missing # of FCF Reflection(s) Below Theta(Min).          1 Note
PLAT912_ALERT_4_G Missing # of FCF Reflections Above STh/L=  0.600         88 Note
PLAT913_ALERT_3_G Missing # of Very Strong Reflections in FCF ....          1 Note
PLAT929_ALERT_5_G No Weight Pars,Obs and Calc R1,wR2,S not Checked          ! Info
PLAT960_ALERT_3_G Number of Intensities with I  <   - 2*sig(I) ...          2 Check
```

---

0 **ALERT level A** = Most likely a serious problem - resolve or explain
0 **ALERT level B** = A potentially serious problem, consider carefully
6 **ALERT level C** = Check. Ensure it is not caused by an omission or oversight
55 **ALERT level G** = General information/check it is not something unexpected

8 ALERT type 1 CIF construction/syntax error, inconsistent or missing data
2 ALERT type 2 Indicator that the structure model may be wrong or deficient
6 ALERT type 3 Indicator that the structure quality may be low
42 ALERT type 4 Improvement, methodology, query or suggestion
3 ALERT type 5 Informative message, check

It is advisable to attempt to resolve as many as possible of the alerts in all categories. Often the minor alerts point to easily fixed oversights, errors and omissions in your CIF or refinement strategy, so attention to these fine details can be worthwhile. In order to resolve some of the more serious problems it may be necessary to carry out additional measurements or structure refinements. However, the purpose of your study may justify the reported deviations and the more serious of these should normally be commented upon in the discussion or experimental section of a paper or in the "special_details" fields of the CIF. checkCIF was carefully designed to identify outliers and unusual parameters, but every test has its limitations and alerts that are not important in a particular case may appear. Conversely, the absence of alerts does not guarantee there are no aspects of the results needing attention. It is up to the individual to critically assess their own results and, if necessary, seek expert advice.

Publication of your CIF in IUCr journals

A basic structural check has been run on your CIF. These basic checks will be run on all CIFs submitted for publication in IUCr journals (*Acta Crystallographica*, *Journal of Applied Crystallography*, *Journal of Synchrotron Radiation*); however, if you intend to submit to *Acta Crystallographica Section C* or *E* or *IUCrData*, you should make sure that full publication checks are run on the final version of your CIF prior to submission.

Publication of your CIF in other journals

Please refer to the *Notes for Authors* of the relevant journal for any special instructions relating to CIF submission.

PLATON version of 28/11/2022; check.def file version of 28/11/2022

Datablock I - ellipsoid plot

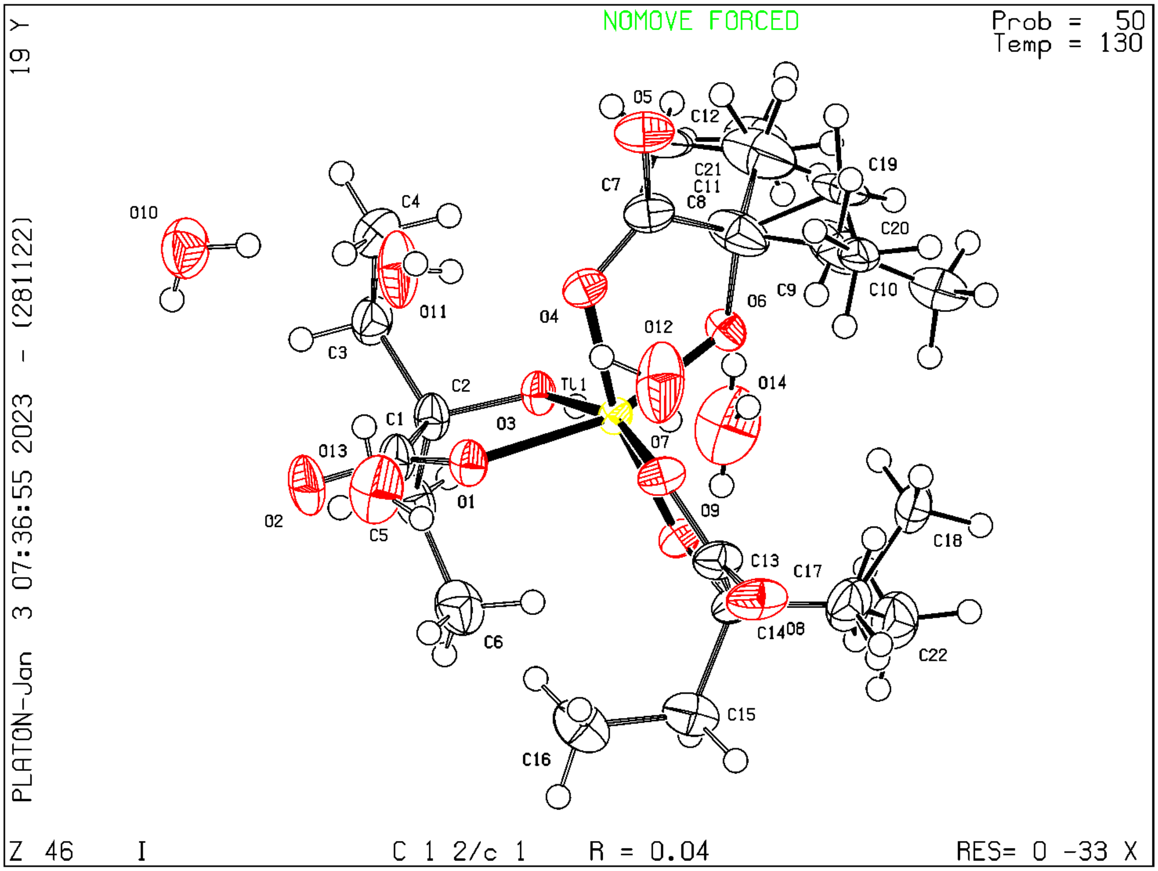

Supplement: Supplementary file 1 [file ijms-24-11865-s001.zip › Final cifs and checkcifs/ki1138telikocheckcif comp4.pdf]
