# Supplementary material for: Structural Speciation of Ti(IV)-(α-Hydroxycarboxylic Acid) Complexes in Metabolism-Related (Patho)Physiology—In Vitro Approaches to (Pre)Adipocyte Differentiation and Mineralization
Source: Int J Mol Sci. 2023 Jul 24;24(14):11865. doi: 10.3390/ijms241411865 (PMC10380816; doi:10.3390/ijms241411865)
Supplement: Supplementary file 1 [file ijms-24-11865-s001.zip › Final cifs and checkcifs/ki487teliko comp3.pdf]

Table S7
Experimental details

Crystal data	
Chemical formula	$C_{21}H_{41.99}N_{2.00}O_{20.00}Ti_{1.00}$
M_r	690.35
Crystal system, space group	Cubic, $P2_13$
Temperature (K)	295
a (Å)	15.1689 (6)
V (Å^3)	3490.3 (4)
Z	4
Radiation type	Mo *K*α
μ (mm^{-1})	0.32
Crystal size (mm)	0.19 × 0.16 × 0.11
Data collection	
Diffractometer	Bruker Kappa Apex2
Absorption correction	Numerical<br>Analytical Absorption (De Meulenaer & Tompa, 1965)
T_{min}, T_{max}	0.95, 0.97
No. of measured, independent and observed [$I > 2.0\sigma(I)$] reflections	13087, 2392, 1927
R_{int}	0.056
$(\sin \theta/\lambda)_{max}$ (Å^{-1})	0.625
Refinement	
$R[F^2 > 2\sigma(F^2)]$, $wR(F^2)$, S	0.053, 0.137, 1.00
No. of reflections	1927
No. of parameters	151
No. of restraints	7
H-atom treatment	H atoms treated by a mixture of independent and constrained refinement
$\Delta\rho_{max}$, $\Delta\rho_{min}$ (e Å^{-3})	0.49, −0.29
Absolute structure	Flack (1983), 1067 Friedel-pairs
Absolute structure parameter	−0.02 (6)

Computer programs: Apex2 (Bruker AXS, 2006), *SUPERFLIP* (Palatinus & Chapuis, 2007), *CRYSTALS* (Betteridge *et al.*, 2003), *CAMERON* (Watkin *et al.*, 1996).

Table S8
Selected geometric parameters (Å, º)

Ti1—O1^{i}	2.039 (3)	O5—C4	1.427 (5)
Ti1—O1^{ii}	2.039 (3)	O6—C5	1.437 (6)
Ti1—O3^{ii}	1.866 (2)	C1—C2	1.511 (5)
Ti1—O3^{i}	1.866 (2)	C2—C3	1.531 (6)
Ti1—O1	2.039 (3)	C2—C7	1.532 (5)
Ti1—O3	1.866 (2)	C3—C4	1.529 (6)
O1—C1	1.270 (5)	C4—C5	1.503 (6)
O2—C1	1.238 (5)	C5—C6	1.530 (6)
O3—C2	1.406 (4)	C6—C7	1.518 (6)
O4—C6	1.416 (6)		
O1^{i}—Ti1—O1^{ii}	83.76 (13)	O2—C1—C2	122.6 (4)
O1^{i}—Ti1—O3^{ii}	104.19 (11)	C1—C2—O3	107.0 (3)
O1^{ii}—Ti1—O3^{ii}	78.57 (11)	C1—C2—C3	109.6 (3)
O1^{i}—Ti1—O3^{i}	78.57 (11)	O3—C2—C3	109.9 (3)
O1^{ii}—Ti1—O3^{i}	159.59 (11)	C1—C2—C7	112.6 (3)
O3^{ii}—Ti1—O3^{i}	95.87 (11)	O3—C2—C7	108.3 (3)
O1^{i}—Ti1—O1	83.76 (13)	C3—C2—C7	109.4 (4)
O1^{ii}—Ti1—O1	83.76 (13)	C2—C3—C4	111.2 (3)
O3^{ii}—Ti1—O1	159.59 (11)	C3—C4—O5	110.3 (4)
O3^{i}—Ti1—O1	104.19 (11)	C3—C4—C5	111.6 (4)
O1^{i}—Ti1—O3	159.59 (11)	O5—C4—C5	110.6 (4)
O1^{ii}—Ti1—O3	104.19 (11)	C4—C5—O6	107.1 (4)
O3^{ii}—Ti1—O3	95.87 (11)	C4—C5—C6	112.0 (4)
O3^{i}—Ti1—O3	95.87 (11)	O6—C5—C6	110.6 (4)
O1—Ti1—O3	78.57 (11)	C5—C6—O4	112.1 (4)
Ti1—O1—C1	116.7 (2)	C5—C6—C7	111.6 (4)
Ti1—O3—C2	121.8 (2)	O4—C6—C7	111.2 (4)
O1—C1—O2	122.3 (4)	C2—C7—C6	110.6 (3)
O1—C1—C2	115.2 (3)		

Symmetry codes: (i) $-z+3/2$, $-x+1$, $y+1/2$; (ii) $-y+1$, $z-1/2$, $-x+3/2$.

Table S9
Hydrogen-bond geometry (Å, º)

D—H···*A*	*D*—H	H···*A*	*D*···*A*	*D*—H···*A*

O7—H72···O6	0.82	2.10	2.922 (6)	179
O7—H71···O8	0.82	2.17	2.987 (6)	178
N3—H32···O4^{i}	0.85	1.95	2.741 (6)	153
N3—H31···O5	0.86	2.00	2.808 (6)	155
N3—H34···N1	0.85	2.15	2.907 (6)	147
N3—H33···O7^{iii}	0.86	2.02	2.883 (6)	177
O9—H91···O4	0.82	2.01	2.825 (6)	179
O9—H92···O5^{ii}	0.82	2.19	3.010 (6)	179
O8—H81···O4^{iv}	0.79	2.30	3.086 (6)	175
O8—H82···O7^{i}	0.82	1.66	2.278 (6)	130
O8—H82···O9^{i}	0.82	2.19	3.011 (6)	176
O6—H97···O1^{v}	0.82	2.49	3.031 (6)	124
O6—H97···O2^{v}	0.82	2.16	2.978 (6)	180
O5—H98···O5^{vi}	0.77	2.16	2.896 (6)	160
O4—H99···O3	0.82	1.85	2.668 (6)	179
O4—H99···C2	0.82	2.32	3.011 (6)	142
N1—H14···O2^{vii}	0.85	2.00	2.793 (6)	155

Symmetry codes: (i) −*z*+3/2, −*x*+1, *y*+1/2; (ii) −*y*+1, *z*−1/2, −*x*+3/2; (iii) *y*+1/2, −*z*+3/2, −*x*+1; (iv) −*x*+1, *y*+1/2, −*z*+3/2; (v) *x*−1/2, −*y*+1/2, −*z*+1; (vi) *y*, *z*, *x*; (vii) *z*+1/2, −*x*+3/2, −*y*+1.

Supporting information

Crystallographic data

Acknowledgements

Funding information

References

43. Bruker Analytical X-ray Systems, Inc. Apex2, Version 2 User Manual, M86-E01078; Bruker Analytical X-ray Systems, Inc: Madison, WI, USA, 2006.

46. Betteridge, P.W.; Carruthers, J.R.; Cooper, R.I.; Prout, K.; Watkin, D.J.J. CRYSTALS version 12: software for guided crystal structure analysis. Appl. Cryst. 2003, 36, 1487. https://doi.org/10.1107/S0021889803021800.

49. De Meulenaer, J.; Tompa, H. The absorption correction in crystal structure analysis. *Acta Cryst.* **1965**, *19*, 1014-1018. https://doi.org/10.1107/S0365110X65004802.
50. Prince, E. *Mathematical Techniques in Crystallography and Materials Science*; Springer: New York, NY, USA, 1982.
51. Watkin, D.J. The control of difficult refinements. *Acta Cryst.* **1994**, A*50*, 411–437. https://doi.org/10.1107/S0108767393012784.

52. Watkin, D.J.; Prout, C.K.; Pearce, L.J. *CAMERON*; Chemical Crystallography Laboratory: Oxford, UK, 1996.
53. Flack, H.D. On enantiomorph-polarity estimation. *Acta Cryst.* **1983**, A*39*, 876–881. https://doi.org/10.1107/S0108767383001762.
