# Supplementary material for: Structural Speciation of Ti(IV)-(α-Hydroxycarboxylic Acid) Complexes in Metabolism-Related (Patho)Physiology—In Vitro Approaches to (Pre)Adipocyte Differentiation and Mineralization
Source: Int J Mol Sci. 2023 Jul 24;24(14):11865. doi: 10.3390/ijms241411865 (PMC10380816; doi:10.3390/ijms241411865)

checkCIF/PLATON report

Structure factors have been supplied for datablock(s) I

THIS REPORT IS FOR GUIDANCE ONLY. IF USED AS PART OF A REVIEW PROCEDURE FOR PUBLICATION, IT SHOULD NOT REPLACE THE EXPERTISE OF AN EXPERIENCED CRYSTALLOGRAPHIC REFEREE.

No syntax errors found. CIF dictionary Interpreting this report

Datablock: I

```
Bond precision:   C-C = 0.0059 A          Wavelength=0.71073

Cell:              a=15.1689(6)      b=15.1689(6)      c=15.1689(6)
                   alpha=90          beta=90           gamma=90
Temperature:       295 K
```

	Calculated	Reported
Volume	3490.3(4)	3490.3(4)
Space group	P 21 3	P 21 3
Hall group	P 2ac 2ab 3	?
Moiety formula	C21 H30 O18 Ti, 2(H4 N), 2(H2 O)	C21 H41.99 N2.00 O20.00 Ti1.00
Sum formula	C21 H41.99 N2 O20 Ti	C21 H41.99 N2.00 O20.00 Ti1.00
Mr	690.32	690.35
Dx,g cm-3	1.314	1.314
Z	4	4
Mu (mm-1)	0.323	0.323
F000	1455.7	1455.7
F000’	1457.85	
h,k,lmax	18,18,18	12,13,18
Nref	2382[1326]	2392
Tmin,Tmax	0.940,0.965	0.950,0.970
Tmin’	0.940	

```
Correction method= # Reported T Limits: Tmin=0.950 Tmax=0.970
AbsCorr = NUMERICAL

Data completeness= 1.80/1.00        Theta(max)= 26.391
```

R(reflections)= 0.0531(1927)

wR2(reflections)= 0.1492(1927)

S = 1.000 Npar= 151

The following ALERTS were generated. Each ALERT has the format
test-name_ALERT_alert-type_alert-level.
Click on the hyperlinks for more details of the test.

Alert level C

```
PLAT042_ALERT_1_C Calc. and Reported MoietyFormula Strings  Differ     Please Check
PLAT076_ALERT_1_C Occupancy      0.333 Less Than 1.0 for Sp.pos  .            N2
PLAT076_ALERT_1_C Occupancy      0.333 Less Than 1.0 for Sp.pos  .           H21
PLAT420_ALERT_2_C D-H Bond Without Acceptor  N1       --H13      .     Please Check
PLAT911_ALERT_3_C Missing FCF Refl Between Thmin & STh/L=    0.600          8 Report
```

Alert level G

```
PLAT002_ALERT_2_G Number of Distance or Angle Restraints on AtSite          6 Note
PLAT007_ALERT_5_G Number of Unrefined Donor-H Atoms .............          17 Report
PLAT093_ALERT_1_G No s.u.'s on H-positions, Refinement Reported as      mixed Check
PLAT300_ALERT_4_G Atom Site Occupancy of Ti1        Constrained at      0.999 Check
PLAT300_ALERT_4_G Atom Site Occupancy of N1         Constrained at      0.666 Check
PLAT300_ALERT_4_G Atom Site Occupancy of H13        Constrained at      0.666 Check
PLAT300_ALERT_4_G Atom Site Occupancy of H14        Constrained at      0.666 Check
PLAT300_ALERT_4_G Atom Site Occupancy of N2         Constrained at      0.333 Check
PLAT300_ALERT_4_G Atom Site Occupancy of H21        Constrained at      0.333 Check
PLAT300_ALERT_4_G Atom Site Occupancy of H24        Constrained at      0.333 Check
PLAT300_ALERT_4_G Atom Site Occupancy of O7         Constrained at        0.2 Check
PLAT300_ALERT_4_G Atom Site Occupancy of H71        Constrained at        0.2 Check
PLAT300_ALERT_4_G Atom Site Occupancy of H72        Constrained at        0.2 Check
PLAT300_ALERT_4_G Atom Site Occupancy of O8         Constrained at        0.2 Check
PLAT300_ALERT_4_G Atom Site Occupancy of H81        Constrained at        0.2 Check
PLAT300_ALERT_4_G Atom Site Occupancy of H82        Constrained at        0.2 Check
PLAT300_ALERT_4_G Atom Site Occupancy of O9         Constrained at      0.266 Check
PLAT300_ALERT_4_G Atom Site Occupancy of H91        Constrained at      0.266 Check
PLAT300_ALERT_4_G Atom Site Occupancy of H92        Constrained at      0.266 Check
PLAT300_ALERT_4_G Atom Site Occupancy of N3         Constrained at      0.333 Check
PLAT300_ALERT_4_G Atom Site Occupancy of H31        Constrained at      0.333 Check
PLAT300_ALERT_4_G Atom Site Occupancy of H32        Constrained at      0.333 Check
PLAT300_ALERT_4_G Atom Site Occupancy of H33        Constrained at      0.333 Check
PLAT300_ALERT_4_G Atom Site Occupancy of H34        Constrained at      0.333 Check
PLAT301_ALERT_3_G Main Residue  Disorder .............(Resd  1  )          7% Note
PLAT302_ALERT_4_G Anion/Solvent/Minor-Residue Disorder (Resd  2  )       100% Note
PLAT302_ALERT_4_G Anion/Solvent/Minor-Residue Disorder (Resd  3  )       100% Note
PLAT302_ALERT_4_G Anion/Solvent/Minor-Residue Disorder (Resd  4  )       100% Note
PLAT302_ALERT_4_G Anion/Solvent/Minor-Residue Disorder (Resd  5  )       100% Note
PLAT302_ALERT_4_G Anion/Solvent/Minor-Residue Disorder (Resd  6  )       100% Note
PLAT302_ALERT_4_G Anion/Solvent/Minor-Residue Disorder (Resd  7  )       100% Note
PLAT304_ALERT_4_G Non-Integer Number of Atoms in ..... (Resd  2  )       3.33 Check
PLAT304_ALERT_4_G Non-Integer Number of Atoms in ..... (Resd  3  )       1.66 Check
PLAT304_ALERT_4_G Non-Integer Number of Atoms in ..... (Resd  4  )       0.60 Check
PLAT304_ALERT_4_G Non-Integer Number of Atoms in ..... (Resd  5  )       0.60 Check
PLAT304_ALERT_4_G Non-Integer Number of Atoms in ..... (Resd  6  )       0.80 Check
PLAT304_ALERT_4_G Non-Integer Number of Atoms in ..... (Resd  7  )       1.66 Check
```

```
PLAT769_ALERT_4_G CIF Embedded explicitly supplied scattering data     Please Note
PLAT791_ALERT_4_G Model has Chirality at C4          (Sohnke SpGr)          R Verify
PLAT791_ALERT_4_G Model has Chirality at C6          (Sohnke SpGr)          R Verify
PLAT808_ALERT_5_G No Parseable SHELXL Style Weighting Scheme Found     Please Check
PLAT860_ALERT_3_G Number of Least-Squares Restraints ............          7 Note
PLAT882_ALERT_1_G No Datum for _diffrn_reflns_av_unetI/netI ......     Please Do !
PLAT929_ALERT_5_G No Weight Pars,Obs and Calc R1,wR2,S not Checked          ! Info
PLAT960_ALERT_3_G Number of Intensities with I  <   - 2*sig(I) ...          9 Check
```

```
   0 ALERT level A = Most likely a serious problem - resolve or explain
   0 ALERT level B = A potentially serious problem, consider carefully
   5 ALERT level C = Check. Ensure it is not caused by an omission or oversight
  45 ALERT level G = General information/check it is not something unexpected

   5 ALERT type 1 CIF construction/syntax error, inconsistent or missing data
   2 ALERT type 2 Indicator that the structure model may be wrong or deficient
   4 ALERT type 3 Indicator that the structure quality may be low
  36 ALERT type 4 Improvement, methodology, query or suggestion
   3 ALERT type 5 Informative message, check
```

It is advisable to attempt to resolve as many as possible of the alerts in all categories. Often the minor alerts point to easily fixed oversights, errors and omissions in your CIF or refinement strategy, so attention to these fine details can be worthwhile. In order to resolve some of the more serious problems it may be necessary to carry out additional measurements or structure refinements. However, the purpose of your study may justify the reported deviations and the more serious of these should normally be commented upon in the discussion or experimental section of a paper or in the "special_details" fields of the CIF. checkCIF was carefully designed to identify outliers and unusual parameters, but every test has its limitations and alerts that are not important in a particular case may appear. Conversely, the absence of alerts does not guarantee there are no aspects of the results needing attention. It is up to the individual to critically assess their own results and, if necessary, seek expert advice.

Publication of your CIF in IUCr journals

A basic structural check has been run on your CIF. These basic checks will be run on all CIFs submitted for publication in IUCr journals (*Acta Crystallographica*, *Journal of Applied Crystallography*, *Journal of Synchrotron Radiation*); however, if you intend to submit to *Acta Crystallographica Section C* or *E* or *IUCrData*, you should make sure that full publication checks are run on the final version of your CIF prior to submission.

Publication of your CIF in other journals

Please refer to the *Notes for Authors* of the relevant journal for any special instructions relating to CIF submission.

PLATON version of 28/11/2022; check.def file version of 28/11/2022

Datablock I - ellipsoid plot

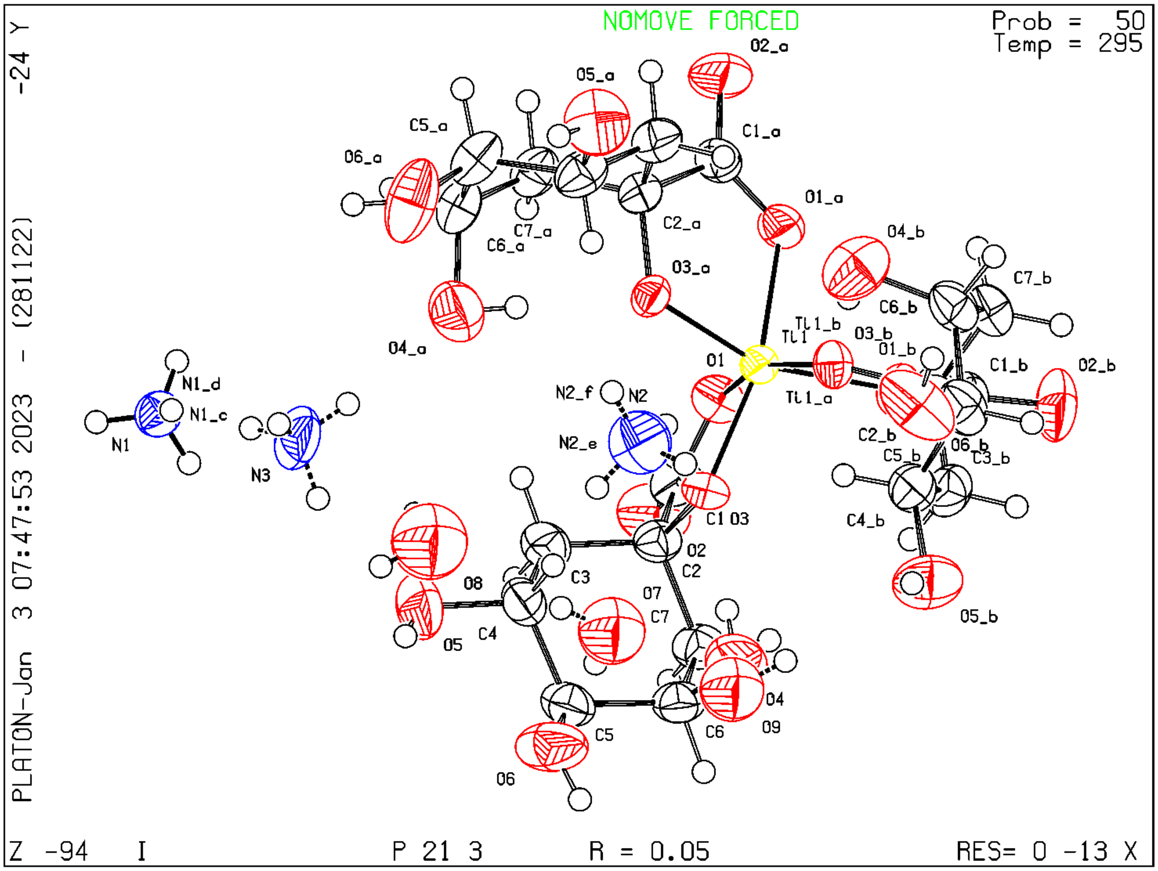

Supplement: Supplementary file 1 [file ijms-24-11865-s001.zip › Final cifs and checkcifs/ki487telikocheckcif comp3.pdf]
