# Supplementary material for: Structural Speciation of Ti(IV)-(α-Hydroxycarboxylic Acid) Complexes in Metabolism-Related (Patho)Physiology—In Vitro Approaches to (Pre)Adipocyte Differentiation and Mineralization
Source: Int J Mol Sci. 2023 Jul 24;24(14):11865. doi: 10.3390/ijms241411865 (PMC10380816; doi:10.3390/ijms241411865)
Supplement: Supplementary file 1 [file ijms-24-11865-s001.zip › Final cifs and checkcifs/ki835bteliko comp1.pdf]

Table S1
Experimental details

Crystal data	
Chemical formula	$C_{24}H_{66}N_4O_{25}Ti_2$
M_r	906.60
Crystal system, space group	Triclinic, *P*-1
Temperature (K)	295
a, *b*, *c* (Å)	10.530 (6), 11.531 (6), 18.946 (11)
α, β, γ (°)	89.09 (3), 89.82 (3), 80.80 (3)
V (Å^3)	2270 (2)
Z	2
Radiation type	Mo *K*α
μ (mm^{-1})	0.43
Crystal size (mm)	0.26 × 0.16 × 0.13
Data collection	
Diffractometer	Bruker Kappa Apex2
Absorption correction	Numerical<br>Analytical Absorption (De Meulenaer & Tompa, 1965)
T_{min}, T_{max}	0.93, 0.95
No. of measured, independent and observed [$I > 2.0\sigma(I)$] reflections	69918, 9847, 8419
R_{int}	0.015
$(\sin \theta/\lambda)_{max}$ (Å^{-1})	0.643
Refinement	
$R[F^2 > 2\sigma(F^2)]$, $wR(F^2)$, S	0.041, 0.062, 1.00
No. of reflections	8419
No. of parameters	499
H-atom treatment	H-atom parameters constrained
$\Delta\rho_{max}$, $\Delta\rho_{min}$ (e Å^{-3})	0.26, −0.34

Computer programs: Apex2 (Bruker AXS, 2006), *SUPERFLIP* (Palatinus & Chapuis, 2007), *CRYSTALS* (Betteridge *et al.*, 2003), *CAMERON* (Watkin *et al.*, 1996).

Table S2
Selected geometric parameters (Å, º)

Ti1—O1	2.0378 (16)	O13—C17	1.281 (2)
Ti1—O3	1.8742 (15)	O14—C17	1.235 (2)
Ti1—O4	2.0644 (16)	O15—C18	1.428 (2)

Ti1—O6	1.8639 (15)	O16—C21	1.281 (2)
Ti1—O7	2.0363 (17)	O17—C21	1.232 (2)
Ti1—O9	1.8703 (15)	O18—C22	1.432 (2)
Ti2—O10	2.0546 (17)	C1—C2	1.528 (3)
Ti2—O12	1.8679 (15)	C2—C3	1.524 (3)
Ti2—O13	2.0414 (17)	C2—C4	1.522 (3)
Ti2—O15	1.8666 (15)	C5—C6	1.542 (3)
Ti2—O16	2.0474 (17)	C6—C7	1.518 (3)
Ti2—O18	1.8817 (15)	C6—C8	1.509 (3)
O1—C1	1.297 (2)	C9—C10	1.533 (3)
O2—C1	1.224 (2)	C10—C11	1.524 (3)
O3—C2	1.430 (2)	C10—C12	1.531 (3)
O4—C5	1.274 (2)	C13—C14	1.511 (3)
O5—C5	1.238 (2)	C14—C15	1.531 (3)
O6—C6	1.420 (2)	C14—C16	1.522 (3)
O7—C9	1.289 (2)	C17—C18	1.535 (3)
O8—C9	1.229 (2)	C18—C19	1.517 (3)
O9—C10	1.431 (2)	C18—C20	1.517 (3)
O10—C13	1.271 (2)	C21—C22	1.529 (3)
O11—C13	1.244 (2)	C22—C23	1.506 (3)
O12—C14	1.424 (2)	C22—C24	1.523 (3)

O1—Ti1—O3	78.95 (6)	C1—C2—C4	109.6 (2)
O1—Ti1—O4	80.72 (7)	O3—C2—C4	109.1 (2)
O3—Ti1—O4	158.42 (6)	C3—C2—C4	112.0 (2)
O1—Ti1—O6	101.93 (7)	O4—C5—O5	122.94 (19)
O3—Ti1—O6	98.05 (7)	O4—C5—C6	115.23 (16)
O4—Ti1—O6	79.15 (7)	O5—C5—C6	121.83 (17)
O1—Ti1—O7	83.54 (7)	C5—C6—O6	106.91 (15)
O3—Ti1—O7	103.48 (8)	C5—C6—C7	109.48 (18)
O4—Ti1—O7	81.22 (8)	O6—C6—C7	108.59 (16)
O6—Ti1—O7	158.44 (6)	C5—C6—C8	110.45 (17)
O1—Ti1—O9	160.26 (5)	O6—C6—C8	109.98 (17)
O3—Ti1—O9	95.52 (8)	C7—C6—C8	111.30 (19)
O4—Ti1—O9	106.06 (6)	O7—C9—O8	122.7 (2)
O6—Ti1—O9	97.59 (7)	O7—C9—C10	113.95 (17)
O7—Ti1—O9	79.33 (6)	O8—C9—C10	123.30 (19)
O10—Ti2—O12	78.46 (6)	C9—C10—O9	106.56 (15)
O10—Ti2—O13	81.99 (8)	C9—C10—C11	108.83 (18)

O12—Ti2—O13	158.67 (6)	O9—C10—C11	108.87 (18)
O10—Ti2—O15	104.53 (7)	C9—C10—C12	111.18 (19)
O12—Ti2—O15	96.88 (7)	O9—C10—C12	110.21 (17)
O13—Ti2—O15	79.81 (7)	C11—C10—C12	111.06 (18)
O10—Ti2—O16	83.22 (7)	O10—C13—O11	122.93 (18)
O12—Ti2—O16	101.00 (7)	O10—C13—C14	116.25 (16)
O13—Ti2—O16	84.96 (8)	O11—C13—C14	120.82 (18)
O15—Ti2—O16	161.65 (6)	C13—C14—O12	106.37 (15)
O10—Ti2—O18	160.75 (6)	C13—C14—C15	110.61 (17)
O12—Ti2—O18	98.69 (8)	O12—C14—C15	109.98 (16)
O13—Ti2—O18	102.58 (7)	C13—C14—C16	109.75 (17)
O15—Ti2—O18	94.70 (7)	O12—C14—C16	107.85 (17)
O16—Ti2—O18	78.62 (6)	C15—C14—C16	112.08 (19)
Ti1—O1—C1	117.38 (12)	O13—C17—O14	122.2 (2)
Ti1—O3—C2	122.08 (12)	O13—C17—C18	115.72 (17)
Ti1—O4—C5	115.81 (12)	O14—C17—C18	122.12 (19)
Ti1—O6—C6	121.39 (12)	C17—C18—O15	106.12 (16)
Ti1—O7—C9	116.65 (12)	C17—C18—C19	112.81 (19)
Ti1—O9—C10	119.33 (11)	O15—C18—C19	108.74 (17)
Ti2—O10—C13	116.23 (12)	C17—C18—C20	107.97 (19)
Ti2—O12—C14	122.39 (11)	O15—C18—C20	109.97 (18)
Ti2—O13—C17	115.67 (13)	C19—C18—C20	111.1 (2)
Ti2—O15—C18	120.59 (12)	O16—C21—O17	123.35 (18)
Ti2—O16—C21	117.47 (12)	O16—C21—C22	115.07 (16)
Ti2—O18—C22	122.02 (11)	O17—C21—C22	121.57 (18)
O1—C1—O2	123.1 (2)	C21—C22—O18	106.71 (16)
O1—C1—C2	114.51 (17)	C21—C22—C23	110.05 (18)
O2—C1—C2	122.42 (19)	O18—C22—C23	109.17 (18)
C1—C2—O3	106.95 (16)	C21—C22—C24	108.65 (18)
C1—C2—C3	110.3 (2)	O18—C22—C24	109.06 (17)
O3—C2—C3	108.72 (19)	C23—C22—C24	113.0 (2)

Table S3
Hydrogen-bond geometry (Å, º)

D—H···*A*	*D*—H	H···*A*	*D*···*A*	*D*—H···*A*
O25—H252···O14^{i}	0.83	2.14	2.909 (3)	153
O25—H251···O20	0.83	2.18	2.958 (3)	156
O26—H251···O20	0.90	2.18	2.909 (3)	138

O26—H261···O14^{i}	0.83	2.59	3.347 (3)	153
O26—H262···O20	0.83	2.16	2.909 (3)	150
O27—H271···O14^{i}	0.83	1.69	2.475 (3)	158
O27—H271···C17^{i}	0.83	2.39	3.168 (3)	155
O25—H272···O20	1.04	2.10	2.958 (3)	137
O27—H272···O20	0.83	2.10	2.899 (3)	160
C7—H72···O27^{ii}	0.96	2.43	3.340 (3)	159
C23—H232···O27	0.96	2.55	3.344 (3)	140
N2—H21···O19^{iii}	0.83	2.01	2.800 (3)	157
N2—H24···O21	0.86	2.20	2.757 (3)	123
N2—H23···O17^{ii}	0.82	1.94	2.749 (3)	166
N3—H273···O23^{iv}	0.83	2.35	3.156 (3)	166
N3—H34···O8	0.83	2.00	2.728 (3)	147
N3—H275···O11^{i}	0.82	2.23	2.785 (3)	126
N3—H275···O24^{v}	0.82	2.32	2.829 (3)	121
N1—H11···O18	0.88	1.93	2.793 (3)	167
N1—H13···O19	0.88	1.98	2.805 (3)	156
N1—H14···O5^{iii}	0.87	1.94	2.789 (3)	169
N1—H12···O21	0.87	2.11	2.958 (3)	165
O19—H282···O5	0.85	1.99	2.831 (3)	169
O19—H281···O14^{i}	0.83	1.91	2.737 (3)	169
O22—H222···O3	0.85	1.98	2.814 (3)	168
O22—H221···O11^{vi}	0.82	2.10	2.775 (3)	139
O21—H212···O15	0.85	2.01	2.829 (3)	160
O21—H211···O1	0.83	2.04	2.832 (3)	160
O24—H283···O22	0.82	2.22	2.908 (3)	141
O23—H285···O6	0.84	2.06	2.856 (3)	158
O23—H286···O10^{vii}	0.85	2.19	2.949 (3)	149
N4—H277···O16^{vii}	0.89	2.49	3.225 (3)	141
N4—H277···O17^{vii}	0.89	2.07	2.867 (3)	149
N4—H277···C21^{vii}	0.89	2.58	3.421 (3)	157
N4—H44···O9	0.91	2.00	2.897 (3)	168
N4—H276···O2^{i}	0.91	1.90	2.797 (3)	168
N4—H278···O24	0.79	2.06	2.836 (3)	165
O20—H279···O4	0.83	2.37	3.043 (3)	138
O20—H279···O7	0.83	2.23	2.941 (3)	143
O20—H280···O12	0.83	1.99	2.803 (3)	167

Symmetry codes: (i) $x-1, y, z$; (ii) $x, y+1, z$; (iii) $-x+1, -y+1, -z+2$; (iv) $x, y-1, z$; (v) $-x, -y+1, -z+1$; (vi) $-x+1, -y+1, -z+1$; (vii) $x-1, y+1, z$.

Supporting information

Crystallographic data

Acknowledgements
Funding information
References

43. Bruker Analytical X-ray Systems, Inc. Apex2, Version 2 User Manual, M86-E01078; Bruker Analytical X-ray Systems, Inc: Madison, WI, USA, 2006.

46. Betteridge, P.W.; Carruthers, J.R.; Cooper, R.I.; Prout, K.; Watkin, D.J.J. CRYSTALS version 12: software for guided crystal structure analysis. Appl. Cryst. 2003, 36, 1487. https://doi.org/10.1107/S0021889803021800.

49. De Meulenaer, J.; Tompa, H. The absorption correction in crystal structure analysis. *Acta Cryst.* **1965**, *19*, 1014-1018. https://doi.org/10.1107/S0365110X65004802.
50. Prince, E. *Mathematical Techniques in Crystallography and Materials Science*; Springer: New York, NY, USA, 1982.
51. Watkin, D.J. The control of difficult refinements. *Acta Cryst.* **1994**, A*50*, 411–437. https://doi.org/10.1107/S0108767393012784.
52. Watkin, D.J.; Prout, C.K.; Pearce, L.J. *CAMERON*; Chemical Crystallography Laboratory: Oxford, UK, 1996.
53. Flack, H.D. On enantiomorph-polarity estimation. *Acta Cryst.* **1983**, A*39*, 876–881. https://doi.org/10.1107/S0108767383001762.
