# Supplementary material for: Structural Speciation of Ti(IV)-(α-Hydroxycarboxylic Acid) Complexes in Metabolism-Related (Patho)Physiology—In Vitro Approaches to (Pre)Adipocyte Differentiation and Mineralization
Source: Int J Mol Sci. 2023 Jul 24;24(14):11865. doi: 10.3390/ijms241411865 (PMC10380816; doi:10.3390/ijms241411865)

checkCIF/PLATON report

Structure factors have been supplied for datablock(s) I

THIS REPORT IS FOR GUIDANCE ONLY. IF USED AS PART OF A REVIEW PROCEDURE FOR PUBLICATION, IT SHOULD NOT REPLACE THE EXPERTISE OF AN EXPERIENCED CRYSTALLOGRAPHIC REFEREE.

No syntax errors found. CIF dictionary Interpreting this report

Datablock: I

Bond precision: C-C = 0.0032 A Wavelength=0.71073

Cell:	a=10.530(6)	b=11.531(6)	c=18.946(11)
	alpha=89.09(3)	beta=89.82(3)	gamma=80.80(3)
Temperature:	295 K		

	Calculated	Reported
Volume	2271(2)	2270(2)
Space group	P -1	P -1
Hall group	-P 1	?
Moiety formula	2(C12 H18 O9 Ti), 7(H2 O), 4(H4 N)	C24 H66 N4 O25 Ti2
Sum formula	C24 H66 N4 O25 Ti2	C24 H66 N4 O25 Ti2
Mr	906.55	906.60
Dx,g cm-3	1.326	1.326
Z	2	2
Mu (mm-1)	0.434	0.434
F000	964.0	964.0
F000'	965.72	
h,k,lmax	13,14,24	13,14,24
Nref	10151	9847
Tmin,Tmax	0.920,0.945	0.930,0.950
Tmin'	0.893	

Correction method= # Reported T Limits: Tmin=0.930 Tmax=0.950
AbsCorr = NUMERICAL

Data completeness= 0.970 Theta(max)= 27.204

R(reflections)= 0.0406(8419) wR2(reflections)= 0.0627(8419)

S = 1.000 Npar= 499

The following ALERTS were generated. Each ALERT has the format
test-name_ALERT_alert-type_alert-level.
Click on the hyperlinks for more details of the test.

Alert level C

```
PLAT042_ALERT_1_C Calc. and Reported MoietyFormula Strings  Differ     Please Check
PLAT420_ALERT_2_C D-H Bond Without Acceptor  N2       --H22      .     Please Check
PLAT420_ALERT_2_C D-H Bond Without Acceptor  N3       --H274     .     Please Check
PLAT911_ALERT_3_C Missing FCF Refl Between Thmin & STh/L=    0.600         42 Report
```

Alert level G

```
PLAT007_ALERT_5_G Number of Unrefined Donor-H Atoms .............         34 Report
PLAT154_ALERT_1_G The s.u.'s on the Cell Angles are Equal ..(Note)       0.03 Degree
PLAT300_ALERT_4_G Atom Site Occupancy of O25        Constrained at        0.5 Check
PLAT300_ALERT_4_G Atom Site Occupancy of H251       Constrained at        0.5 Check
PLAT300_ALERT_4_G Atom Site Occupancy of H252       Constrained at        0.5 Check
PLAT300_ALERT_4_G Atom Site Occupancy of O26        Constrained at       0.25 Check
PLAT300_ALERT_4_G Atom Site Occupancy of H261       Constrained at       0.25 Check
PLAT300_ALERT_4_G Atom Site Occupancy of H262       Constrained at       0.25 Check
PLAT300_ALERT_4_G Atom Site Occupancy of O27        Constrained at       0.25 Check
PLAT300_ALERT_4_G Atom Site Occupancy of H271       Constrained at       0.25 Check
PLAT300_ALERT_4_G Atom Site Occupancy of H272       Constrained at       0.25 Check
PLAT302_ALERT_4_G Anion/Solvent/Minor-Residue Disorder (Resd  9  )       100% Note
PLAT302_ALERT_4_G Anion/Solvent/Minor-Residue Disorder (Resd 14  )       100% Note
PLAT302_ALERT_4_G Anion/Solvent/Minor-Residue Disorder (Resd 15  )       100% Note
PLAT304_ALERT_4_G Non-Integer Number of Atoms in ..... (Resd  9  )       1.50 Check
PLAT304_ALERT_4_G Non-Integer Number of Atoms in ..... (Resd 14  )       0.75 Check
PLAT304_ALERT_4_G Non-Integer Number of Atoms in ..... (Resd 15  )       0.75 Check
PLAT769_ALERT_4_G CIF Embedded explicitly supplied scattering data     Please Note
PLAT794_ALERT_5_G Tentative Bond Valency for Ti1       (IV)      .       4.20 Info
PLAT794_ALERT_5_G Tentative Bond Valency for Ti2       (IV)      .       4.17 Info
PLAT808_ALERT_5_G No Parseable SHELXL Style Weighting Scheme Found     Please Check
PLAT882_ALERT_1_G No Datum for _diffrn_reflns_av_unetI/netI ......     Please Do !
PLAT912_ALERT_4_G Missing # of FCF Reflections Above STh/L=  0.600        289 Note
PLAT913_ALERT_3_G Missing # of Very Strong Reflections in FCF ....          3 Note
PLAT929_ALERT_5_G No Weight Pars,Obs and Calc R1,wR2,S not Checked          ! Info
```

0 **ALERT level A** = Most likely a serious problem - resolve or explain
0 **ALERT level B** = A potentially serious problem, consider carefully
4 **ALERT level C** = Check. Ensure it is not caused by an omission or oversight
25 **ALERT level G** = General information/check it is not something unexpected

3 ALERT type 1 CIF construction/syntax error, inconsistent or missing data
2 ALERT type 2 Indicator that the structure model may be wrong or deficient
2 ALERT type 3 Indicator that the structure quality may be low
17 ALERT type 4 Improvement, methodology, query or suggestion
5 ALERT type 5 Informative message, check

It is advisable to attempt to resolve as many as possible of the alerts in all categories. Often the minor alerts point to easily fixed oversights, errors and omissions in your CIF or refinement strategy, so attention to these fine details can be worthwhile. In order to resolve some of the more serious problems it may be necessary to carry out additional measurements or structure refinements. However, the purpose of your study may justify the reported deviations and the more serious of these should normally be commented upon in the discussion or experimental section of a paper or in the "special_details" fields of the CIF. checkCIF was carefully designed to identify outliers and unusual parameters, but every test has its limitations and alerts that are not important in a particular case may appear. Conversely, the absence of alerts does not guarantee there are no aspects of the results needing attention. It is up to the individual to critically assess their own results and, if necessary, seek expert advice.

Publication of your CIF in IUCr journals

A basic structural check has been run on your CIF. These basic checks will be run on all CIFs submitted for publication in IUCr journals (*Acta Crystallographica*, *Journal of Applied Crystallography*, *Journal of Synchrotron Radiation*); however, if you intend to submit to *Acta Crystallographica Section C* or *E* or *IUCrData*, you should make sure that full publication checks are run on the final version of your CIF prior to submission.

Publication of your CIF in other journals

Please refer to the *Notes for Authors* of the relevant journal for any special instructions relating to CIF submission.

PLATON version of 28/11/2022; check.def file version of 28/11/2022

Datablock I - ellipsoid plot

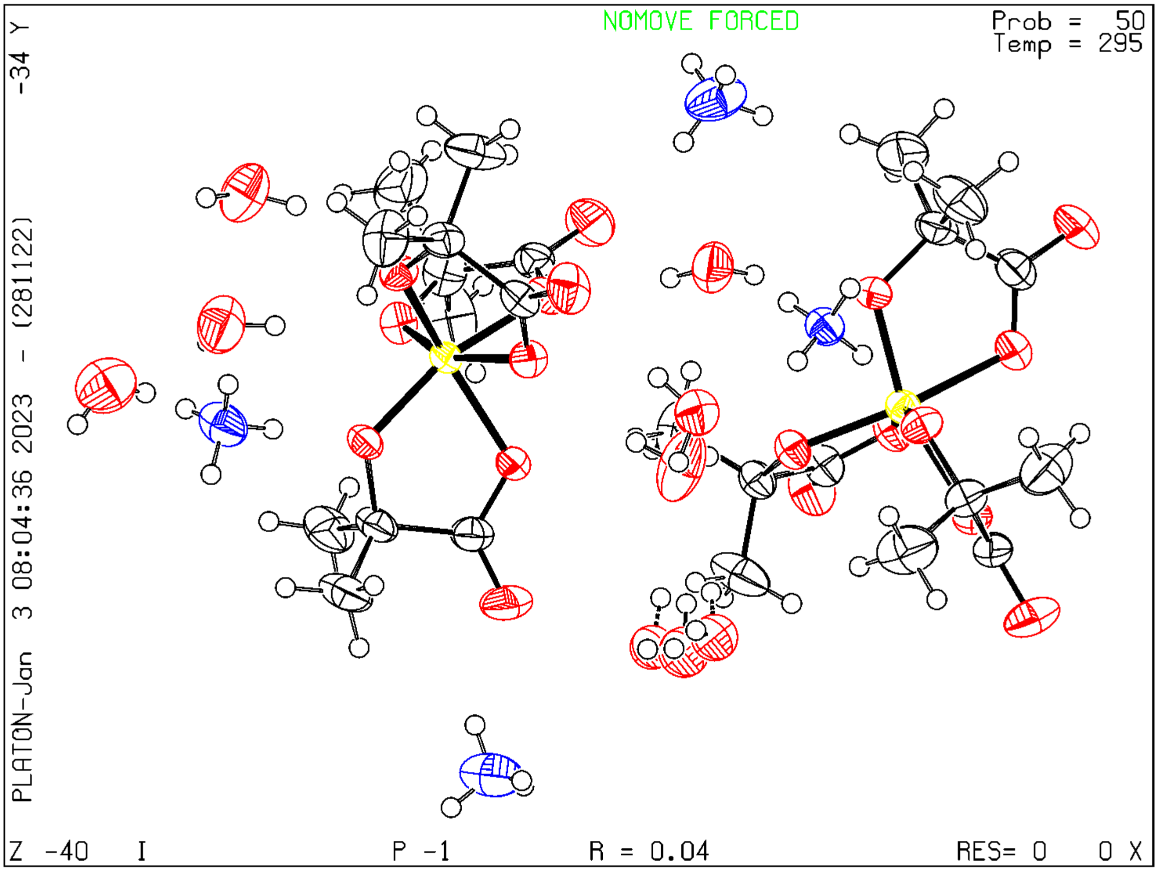

Supplement: Supplementary file 1 [file ijms-24-11865-s001.zip › Final cifs and checkcifs/ki835btelikocheckcif comp1.pdf]
