# Supplementary material for: Structural Speciation of Ti(IV)-(α-Hydroxycarboxylic Acid) Complexes in Metabolism-Related (Patho)Physiology—In Vitro Approaches to (Pre)Adipocyte Differentiation and Mineralization
Source: Int J Mol Sci. 2023 Jul 24;24(14):11865. doi: 10.3390/ijms241411865 (PMC10380816; doi:10.3390/ijms241411865)
Supplement: Supplementary file 1 [file ijms-24-11865-s001.zip › Final cifs and checkcifs/ki835gteliko comp2.pdf]

Table S4
Experimental details

Crystal data	
Chemical formula	$C_{28}H_{66}N_{12}O_{21}Ti_2$
M_r	1002.70
Crystal system, space group	Monoclinic, $P2_1/n$
Temperature (K)	295
a, b, c (Å)	21.458 (7), 10.212 (3), 23.248 (7)
β (°)	94.828 (8)
V (Å^3)	5076 (3)
Z	4
Radiation type	Mo $K\alpha$
μ (mm^{-1})	0.39
Crystal size (mm)	0.22 × 0.13 × 0.11
Data collection	
Diffractometer	Bruker Kappa Apex2
Absorption correction	Numerical<br>Analytical Absorption (De Meulenaer & Tompa, 1965)
T_{min}, T_{max}	0.95, 0.96
No. of measured, independent and observed [$I > 2.0\sigma(I)$] reflections	63319, 9628, 6040
R_{int}	0.057
(sin θ/λ)$_{max}$ (Å^{-1})	0.613
Refinement	
$R[F^2 > 2\sigma(F^2)]$, $wR(F^2)$, S	0.052, 0.088, 1.00
No. of reflections	6040
No. of parameters	568
H-atom treatment	H-atom parameters constrained
$\Delta\rho_{max}$, $\Delta\rho_{min}$ (e Å^{-3})	0.53, −0.39

Computer programs: Apex2 (Bruker AXS, 2006), *SUPERFLIP* (Palatinus & Chapuis, 2007), *CRYSTALS* (Betteridge *et al.*, 2003), *CAMERON* (Watkin *et al.*, 1996).

Table S5
Selected geometric parameters (Å, º)

Ti1—O1	2.065 (2)	C1—C2	1.536 (4)
Ti1—O3	1.856 (2)	C2—C3	1.517 (5)
Ti1—O4	2.070 (2)	C2—C4	1.509 (5)

Ti1—O6	1.861 (2)	C5—C6	1.520 (5)
Ti1—O7	2.052 (2)	C6—C7	1.562 (6)
Ti1—O9	1.860 (2)	C6—C8	1.476 (6)
Ti2—O10	2.048 (2)	C9—C10	1.526 (4)
Ti2—O12	1.878 (2)	C10—C11	1.505 (5)
Ti2—O13	2.061 (2)	C10—C12	1.532 (5)
Ti2—O15	1.874 (2)	C13—C14	1.530 (5)
Ti2—O16	2.072 (2)	C14—C15	1.504 (5)
Ti2—O18	1.827 (2)	C14—C16	1.505 (5)
O1—C1	1.291 (4)	C17—C18	1.523 (5)
O2—C1	1.225 (4)	C18—C19	1.540 (5)
O3—C2	1.427 (4)	C18—C20	1.531 (5)
O4—C5	1.289 (4)	C21—C22	1.539 (4)
O5—C5	1.233 (4)	C22—C23	1.528 (5)
O6—C6	1.405 (4)	C22—C24	1.547 (5)
O7—C9	1.271 (4)	C25—N1	1.324 (4)
O8—C9	1.244 (4)	C25—N2	1.320 (4)
O9—C10	1.422 (4)	C25—N3	1.307 (4)
O10—C13	1.306 (4)	C26—N4	1.295 (5)
O11—C13	1.223 (4)	C26—N5	1.313 (5)
O12—C14	1.425 (4)	C26—N6	1.310 (5)
O13—C17	1.283 (4)	C27—N7	1.309 (4)
O14—C17	1.228 (4)	C27—N8	1.307 (4)
O15—C18	1.386 (4)	C27—N9	1.323 (4)
O16—C21	1.298 (4)	C28—N10	1.308 (5)
O17—C21	1.238 (4)	C28—N11	1.339 (4)
O18—C22	1.410 (4)	C28—N12	1.293 (5)
O1—Ti1—O3	79.17 (9)	C5—C6—O6	108.7 (3)
O1—Ti1—O4	80.80 (8)	C5—C6—C7	107.5 (3)
O3—Ti1—O4	159.23 (9)	O6—C6—C7	107.6 (3)
O1—Ti1—O6	100.37 (10)	C5—C6—C8	111.3 (3)
O3—Ti1—O6	99.60 (10)	O6—C6—C8	110.2 (3)
O4—Ti1—O6	78.67 (9)	C7—C6—C8	111.4 (4)
O1—Ti1—O7	81.86 (9)	O7—C9—O8	124.3 (3)
O3—Ti1—O7	98.51 (9)	O7—C9—C10	115.1 (3)
O4—Ti1—O7	83.97 (8)	O8—C9—C10	120.5 (3)
O6—Ti1—O7	161.85 (9)	C9—C10—O9	107.1 (2)
O1—Ti1—O9	160.92 (9)	C9—C10—C11	109.8 (3)

O3—Ti1—O9	101.20 (9)	O9—C10—C11	109.8 (3)
O4—Ti1—O9	99.53 (9)	C9—C10—C12	108.6 (3)
O6—Ti1—O9	98.37 (10)	O9—C10—C12	108.3 (3)
O7—Ti1—O9	79.22 (9)	C11—C10—C12	113.1 (3)
O10—Ti2—O12	78.78 (9)	O10—C13—O11	123.5 (3)
O10—Ti2—O13	80.55 (9)	O10—C13—C14	113.6 (3)
O12—Ti2—O13	100.63 (10)	O11—C13—C14	122.9 (3)
O10—Ti2—O15	158.26 (10)	C13—C14—O12	107.4 (3)
O12—Ti2—O15	99.05 (10)	C13—C14—C15	109.3 (3)
O13—Ti2—O15	78.60 (9)	O12—C14—C15	109.7 (3)
O10—Ti2—O16	82.84 (9)	C13—C14—C16	107.5 (3)
O12—Ti2—O16	160.96 (9)	O12—C14—C16	109.5 (3)
O13—Ti2—O16	81.08 (9)	C15—C14—C16	113.3 (3)
O15—Ti2—O16	99.87 (9)	O13—C17—O14	122.9 (3)
O10—Ti2—O18	99.39 (10)	O13—C17—C18	113.8 (3)
O12—Ti2—O18	99.36 (10)	O14—C17—C18	123.3 (3)
O13—Ti2—O18	159.58 (9)	C17—C18—O15	109.1 (3)
O15—Ti2—O18	102.29 (10)	C17—C18—C19	108.5 (3)
O16—Ti2—O18	78.67 (9)	O15—C18—C19	110.4 (3)
Ti1—O1—C1	116.30 (19)	C17—C18—C20	109.6 (3)
Ti1—O3—C2	122.42 (16)	O15—C18—C20	108.0 (3)
Ti1—O4—C5	116.43 (19)	C19—C18—C20	111.3 (3)
Ti1—O6—C6	122.1 (2)	O16—C21—O17	122.8 (3)
Ti1—O7—C9	116.42 (19)	O16—C21—C22	114.9 (3)
Ti1—O9—C10	121.64 (17)	O17—C21—C22	122.3 (3)
Ti2—O10—C13	117.3 (2)	C21—C22—O18	105.9 (3)
Ti2—O12—C14	121.67 (19)	C21—C22—C23	110.9 (3)
Ti2—O13—C17	116.7 (2)	O18—C22—C23	110.4 (3)
Ti2—O15—C18	121.84 (19)	C21—C22—C24	107.1 (3)
Ti2—O16—C21	115.50 (18)	O18—C22—C24	109.9 (3)
Ti2—O18—C22	124.87 (18)	C23—C22—C24	112.5 (3)
O1—C1—O2	123.1 (3)	N1—C25—N2	119.8 (3)
O1—C1—C2	114.5 (3)	N1—C25—N3	118.6 (3)
O2—C1—C2	122.5 (3)	N2—C25—N3	121.5 (3)
C1—C2—O3	107.1 (2)	N4—C26—N5	119.0 (4)
C1—C2—C3	108.8 (3)	N4—C26—N6	121.0 (4)
O3—C2—C3	109.1 (3)	N5—C26—N6	120.0 (4)
C1—C2—C4	109.9 (3)	N7—C27—N8	120.9 (3)

O3—C2—C4	109.4 (3)	N7—C27—N9	118.9 (3)
C3—C2—C4	112.4 (3)	N8—C27—N9	120.2 (3)
O4—C5—O5	123.3 (3)	N10—C28—N11	117.6 (4)
O4—C5—C6	113.9 (3)	N10—C28—N12	122.4 (3)
O5—C5—C6	122.8 (3)	N11—C28—N12	119.9 (3)

Table S6
Hydrogen-bond geometry (Å, º)

D—H···*A*	*D*—H	H···*A*	*D*···*A*	*D*—H···*A*
O19—H259···O15^{i}	0.82	2.06	2.844 (5)	160
O19—H258···O6	0.83	2.24	2.855 (5)	131
O20—H256···O19	0.83	2.46	3.277 (5)	168
O20—H257···O12^{i}	0.83	2.04	2.763 (5)	145
N2—H21···O8^{ii}	0.86	2.18	2.933 (5)	146
N2—H22···O3	0.84	2.18	2.942 (5)	150
N3—H251···O19	0.86	2.08	2.921 (5)	165
N3—H250···O17^{iii}	0.86	2.08	2.941 (5)	173
N1—H11···O8^{ii}	0.87	2.25	2.977 (5)	141
N1—H12···O16^{iii}	0.88	2.02	2.883 (5)	165
N11—H253···O20^{i}	0.85	2.08	2.858 (5)	153
N11—H252···O5^{iv}	0.85	2.16	2.948 (5)	153
N10—H101···O11^{v}	0.86	2.58	3.291 (5)	140
N10—H102···O2	0.86	2.12	2.980 (5)	176
N12—H254···O5^{iv}	0.86	2.35	3.091 (5)	145
N12—H255···O1	0.86	2.05	2.887 (5)	165
O21—H212···O17^{iii}	0.82	2.21	3.008 (5)	165
O21—H211···O9	0.82	2.20	2.985 (5)	158
N8—H246···O5^{vi}	0.87	2.15	2.992 (5)	164
N8—H247···O2	0.86	2.10	2.882 (5)	152
N9—H91···O13^{v}	0.86	2.06	2.914 (5)	172
N7—H245···O14^{v}	0.87	2.04	2.904 (5)	171
N7—H244···O4^{vi}	0.85	2.33	2.980 (5)	133
N4—H249···O21^{ii}	0.86	2.27	3.064 (5)	153
N4—H248···O10^{v}	0.87	2.10	2.879 (5)	149
N5—H51···O21^{ii}	0.86	2.37	3.141 (5)	149
N5—H52···O8	0.88	2.07	2.914 (5)	162
N6—H61···O11^{v}	0.86	2.13	2.971 (5)	166
N6—H62···O14^{vii}	0.87	2.30	2.913 (5)	128

Symmetry codes: (i) $-x+1, -y+1, -z+1$; (ii) $-x+1/2, y+1/2, -z+3/2$; (iii) $x-1, y, z$; (iv) $-x+1, -y, -z+1$; (v) $-x+3/2, y+1/2, -z+3/2$; (vi) $x, y+1, z$; (vii) $-x+3/2, y-1/2, -z+3/2$.

Supporting information

Crystallographic data

Acknowledgements
Funding information
References

43. Bruker Analytical X-ray Systems, Inc. Apex2, Version 2 User Manual, M86-E01078; Bruker Analytical X-ray Systems, Inc: Madison, WI, USA, 2006.

46. Betteridge, P.W.; Carruthers, J.R.; Cooper, R.I.; Prout, K.; Watkin, D.J.J. CRYSTALS version 12: software for guided crystal structure analysis. Appl. Cryst. 2003, 36, 1487. https://doi.org/10.1107/S0021889803021800.

49. De Meulenaer, J.; Tompa, H. The absorption correction in crystal structure analysis. *Acta Cryst.* **1965**, *19*, 1014-1018. https://doi.org/10.1107/S0365110X65004802.
50. Prince, E. *Mathematical Techniques in Crystallography and Materials Science*; Springer: New York, NY, USA, 1982.
51. Watkin, D.J. The control of difficult refinements. *Acta Cryst.* **1994**, A*50*, 411–437. https://doi.org/10.1107/S0108767393012784.
52. Watkin, D.J.; Prout, C.K.; Pearce, L.J. *CAMERON*; Chemical Crystallography Laboratory: Oxford, UK, 1996.
53. Flack, H.D. On enantiomorph-polarity estimation. *Acta Cryst.* **1983**, A*39*, 876–881. https://doi.org/10.1107/S0108767383001762.
