# Supplementary material for: Structural Speciation of Ti(IV)-(α-Hydroxycarboxylic Acid) Complexes in Metabolism-Related (Patho)Physiology—In Vitro Approaches to (Pre)Adipocyte Differentiation and Mineralization
Source: Int J Mol Sci. 2023 Jul 24;24(14):11865. doi: 10.3390/ijms241411865 (PMC10380816; doi:10.3390/ijms241411865)
Supplement: Supplementary file 1 [file ijms-24-11865-s001.zip › Supplementary Information.pdf]

Supplementary Information

Structural speciation of Ti(IV)-(α-hydroxycarboxylic acid) complexes in metabolism-related (patho)physiology. *In vitro* approaches to (pre)adipocyte differentiation and mineralization.

Olga Tsave[1], Catherine Iordanidou[1], Antonios Hatzidimitriou[2], Maria P. Yavropoulou[3], Eva N. Kassi[4,5], Narjes Nasiri-Ansari[4], Catherine Gabriel[1], Athanasios Salifoglou[1,*]

***** Author to whom correspondence should be addressed.

Tel: +30-2310-996-179, E-mail: salif@auth.gr

[1] Laboratory of Inorganic Chemistry and Advanced Materials, School of Chemical Engineering, Aristotle University of Thessaloniki, Thessaloniki 54124, Greece

[2] Laboratory of Inorganic Chemistry, School of Chemistry, Aristotle University of Thessaloniki, Thessaloniki 54124, Greece

[3] Endocrinology Unit, 1st Department of Propaedeutic and Internal Medicine, Medical School, National and Kapodistrian University of Athens, Athens 11527, Greece.

[4] Department of Biological Chemistry, Medical School, National and Kapodistrian University of Athens, Athens 11527, Greece.

[5] Endocrine Oncology Unit, 1st Department of Propaedeutic and Internal Medicine, Laiko Hospital, National and Kapodistrian University of Athens, Athens 11527, Greece.

Figure Captions

Figure S1: ESI-MS spectrum of compound **1** in water (positive mode). Full scan spectrum, and simulated spectrum: $M_1 = [Ti(L_1)_3]$, ($m/z = 357.0683$, $z = 1$, $[M_1H_3]^+$).

Figure S2: ESI-MS spectrum of compound **2** in water (positive mode). Full scan spectrum, and simulated spectrum: $M_2 = [Ti(L_1)_3((CH_6N_3)_2)]$, ($m/z = 475.1627$, $z = 1$, $[M_2H]^+$).

Figure S3: ESI-MS spectrum of compound **3** in water (positive mode). Full scan spectrum, and simulated spectrum: $M_{3\_1} = [Ti(L_2)_3]$, ($m/z = 621.1262$, $z = 1$, $[M_{3\_1}H_3]^+$) and $M_{3\_2} = [Ti(L_2)_2]$, ($m/z = 429.0590$, $z = 1$, $[M_{3\_2}H]^+$).

Figure S4: ESI-MS spectrum of compound **4** in water (positive mode). Full scan spectrum, and simulated spectrum: $M_4 = [Ti(L_3H)_2(L_3)]$, ($m/z = 479.1509$, $z = 1$, $[M_4K]^+$).

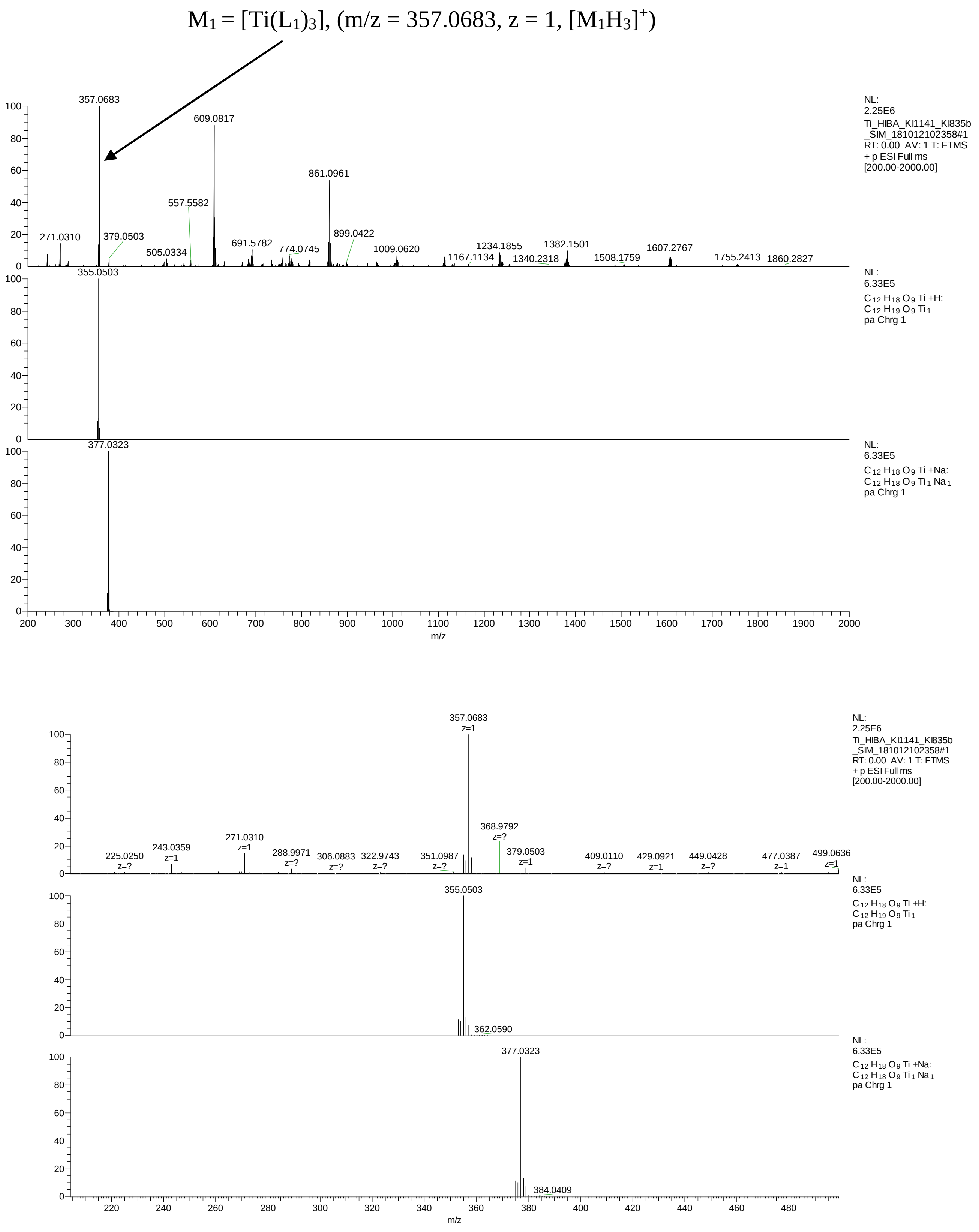
M1 = [Ti(L1)3], (m/z = 357.0683, z = 1, [M1H3]+)
357.0683
609.0817
861.0961
557.5582
271.0310
379.0503
505.0334
691.5782
774.0745
899.0422
1009.0620
1167.1134
1234.1855
1340.2318
1382.1501
1508.1759
1607.2767
1755.2413
1860.2827
NL:
2.25E6
Ti_HIBA_KI1141_KI835b
_SIM_181012102358#1
RT: 0.00 AV: 1 T: FTMS
+ p ESI Full ms
[200.00-2000.00]
355.0503
NL:
6.33E5
C 12 H18 O9 Ti +H:
C 12 H19 O9 Ti 1
pa Chrg 1
377.0323
NL:
6.33E5
C 12 H18 O9 Ti +Na:
C 12 H18 O9 Ti 1 Na 1
pa Chrg 1
m/z
357.0683 z=1
225.0250 z=?
243.0359 z=1
271.0310 z=1
288.9971 z=?
306.0883 z=?
322.9743 z=?
351.0987 z=?
368.9792 z=?
379.0503 z=1
409.0110 z=?
429.0921 z=1
449.0428 z=?
477.0387 z=1
499.0636 z=1
355.0503
362.0590
377.0323
384.0409
m/z

Figure S1

$M_2 = [Ti(L_1)_3((CH_6N_3)_2)]$, ($m/z$ = 475.1627, z = 1, $[M_2H]^+$)

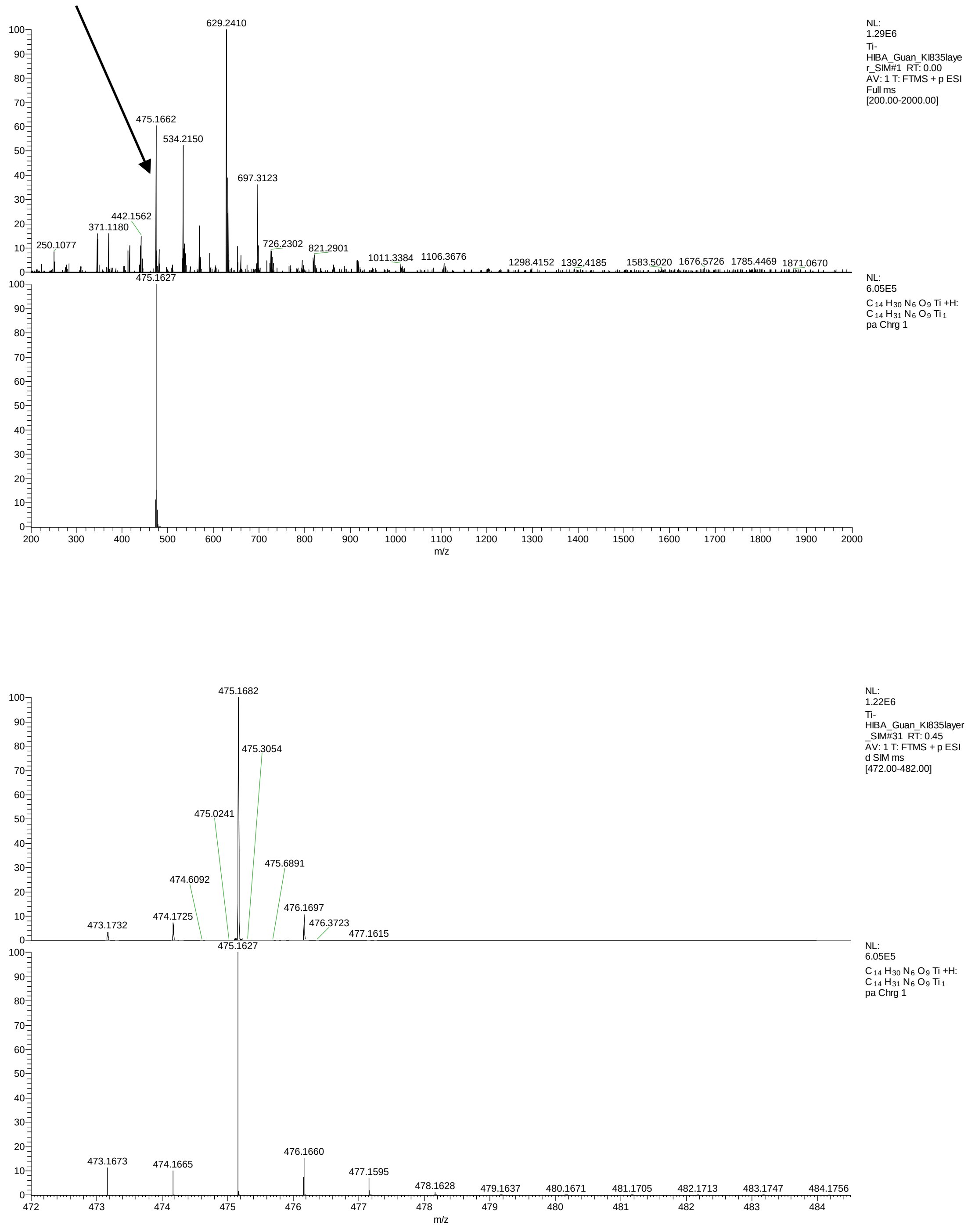

Figure S2

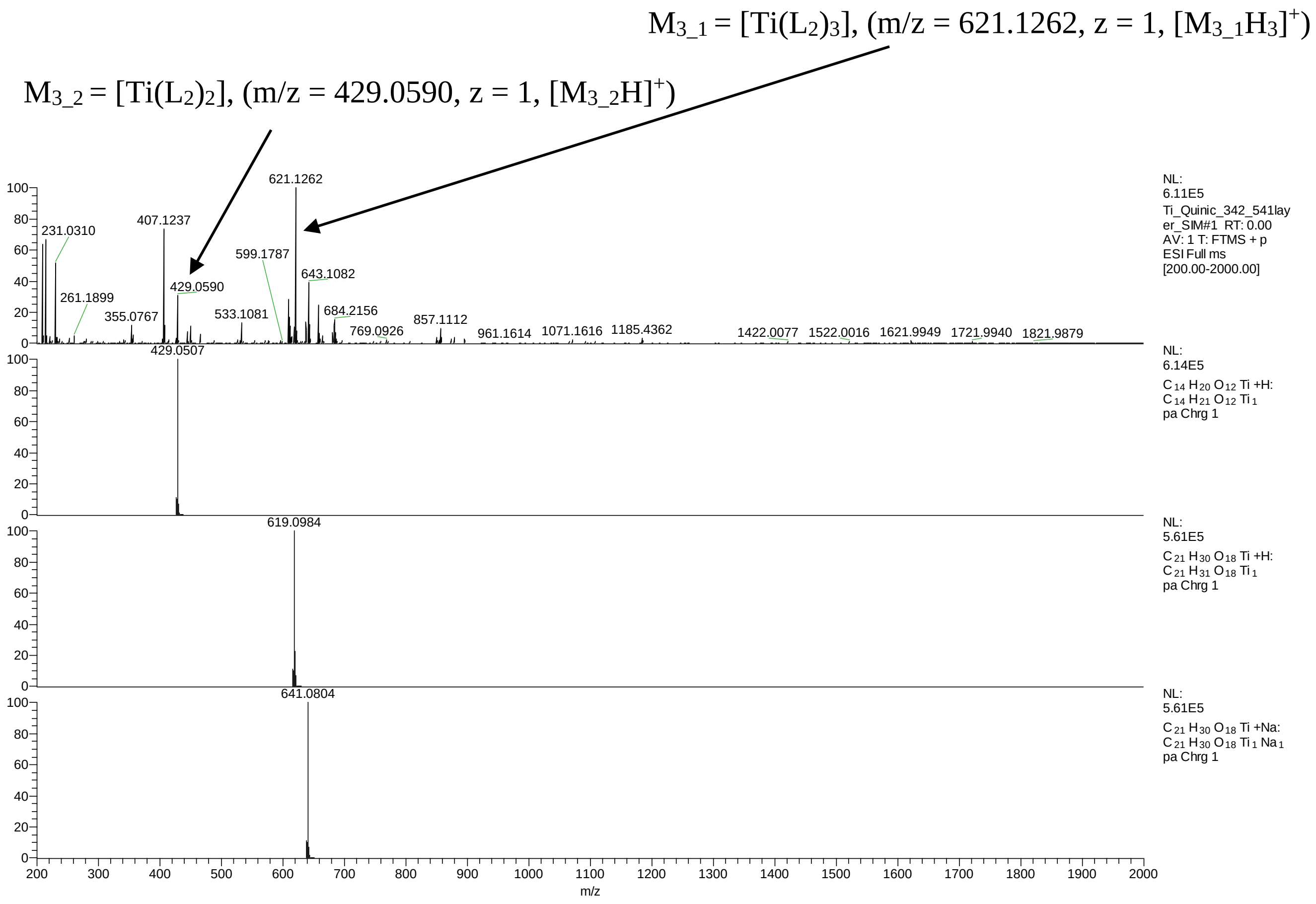

M3_1 = [Ti(L2)3], (m/z = 621.1262, z = 1, [M3_1H3]+)
M3_2 = [Ti(L2)2], (m/z = 429.0590, z = 1, [M3_2H]+)
621.1262
231.0310
407.1237
599.1787
643.1082
429.0590
261.1899
684.2156
355.0767
533.1081
857.1112
769.0926
961.1614
1071.1616
1185.4362
1422.0077
1522.0016
1621.9949
1721.9940
1821.9879
NL: 6.11E5
Ti_Quinic_342_541layer_SIM#1 RT: 0.00
AV: 1 T: FTMS + p ESI Full ms [200.00-2000.00]
429.0507
NL: 6.14E5
C14 H20 O12 Ti +H: C14 H21 O12 Ti1 pa Chrg 1
619.0984
NL: 5.61E5
C21 H30 O18 Ti +H: C21 H31 O18 Ti1 pa Chrg 1
641.0804
NL: 5.61E5
C21 H30 O18 Ti +Na: C21 H30 O18 Ti1 Na1 pa Chrg 1
m/z

Figure S3

M_4=[Ti(L_3H)$_2$(L_3)], (m/z= 479.1569, z=1, [M_4K]$^+$)

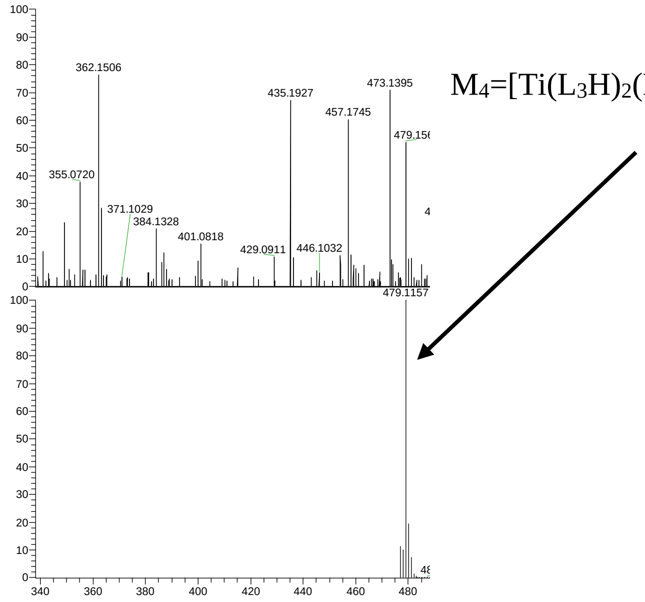

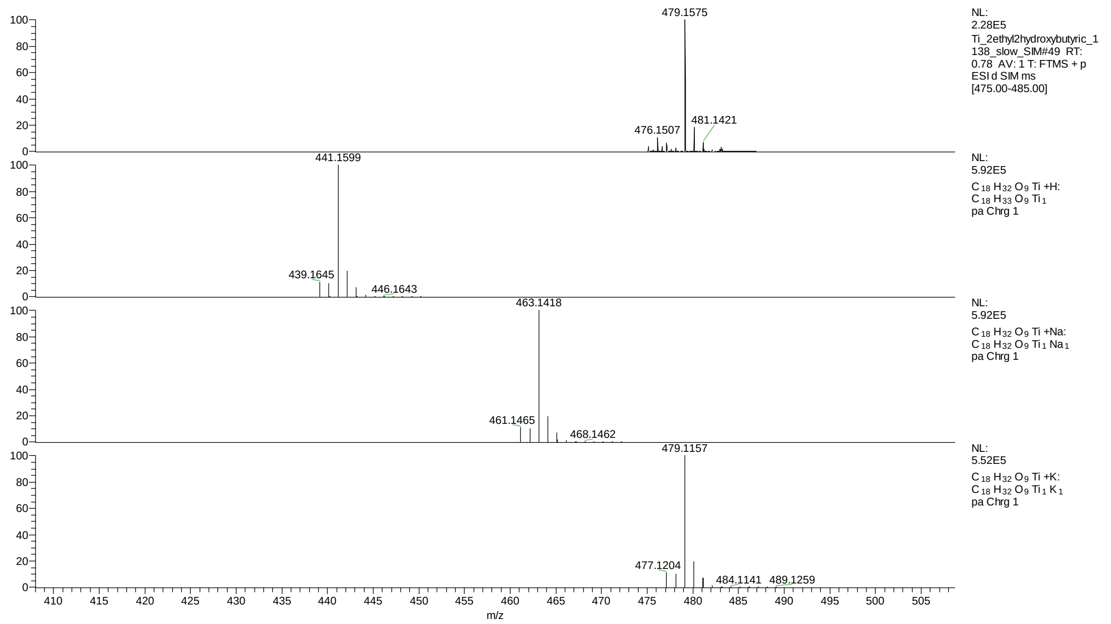

Figure S4
